# Evasion of humoral immune responses by a key mutational region of the S2 subunit in PEDV variants

**DOI:** 10.1128/mbio.00026-26

**Published:** 2026-03-23

**Authors:** Shiyu Liu, Gege Zhang, Jingyuan Xie, Boshui Yuan, Weilu Guo, Yunchuan Li, Rongli Guo, Min Sun, Mi Hu, Yongxiang Zhao, Fei Liu, Qi Peng, Bin Li, Baochao Fan

**Affiliations:** 1 College of Veterinary Medicine, Nanjing Agricultural University 261674, Nanjing, Jiangsu, China; 2 Key Laboratory of Veterinary Biological Engineering and Technology, Institute of Veterinary Medicine, Jiangsu Academy of Agricultural Sciences 668638 , Nanjing, China; 3 Jiangsu Key Laboratory for Food Quality and Safety-State Key Laboratory Cultivation Base of Ministry of Science and Technology, Nanjing, China; 4 Taizhou Polytechnic College 164417 , Taizhou, China; 5 Institute of Pathogenic Microorganism, Jiangxi Agricultural University 91595, Nanchang, China; 6 Jiangsu Co-innovation Center for Prevention and Control of Important Animal Infectious Diseases and Zoonoses, Yangzhou University 38043, Yangzhou, China; Huazhong Agricultural University, Wuhan, Hubei, China

**Keywords:** PEDV, immune evasion, S protein, pathogenicity, membrane fusion

## Abstract

Since 2010, millions of piglets have died from porcine epidemic diarrhea virus (PEDV) variant strains. Compared with classical strains, variants exhibit enhanced virulence and immune evasion capacity, rendering classical strain-based vaccines poorly effective. However, the critical mutants responsible for increased pathogenicity and immune evasion remain unclear. This study aims to identify the key mutations that drive humoral immune evasion in PEDV variants and to elucidate further the underlying molecular mechanisms. Cross-neutralization assays and recombinant virus screening identified the 894–993 amino acid (aa) mutation region of the S2 subunit as a key determinant of humoral immune evasion in variants. The challenge experiments in piglets demonstrated that the 894–993 aa mutation region plays a critical role in determining variants’ virulence. Subsequent vaccination-challenge experiments further clarified the pivotal contribution of the 894–993 aa mutation region in the humoral immune evasion *in vivo*. In addition, the 894–993 aa region determines the membrane fusion characteristics, enabling variants to resist neutralizing sera through cell-to-cell transmission. Moreover, structural analyses revealed that mutations in this region altered the surface electrostatic potential and increased hydrophobicity and rigidity of the S protein. The findings of this study are the first to demonstrate that the 894–993 aa mutation region of the S2 subunit drives humoral immune evasion, modulates the virulence and membrane fusion of PEDV variant strains, and provides a theoretical foundation for the formulation of new prevention and control strategies.

**IMPORTANCE:**

Porcine epidemic diarrhea virus (PEDV) variant strains pose a serious threat to piglet health worldwide. Despite the availability of commercial vaccines developed based on classical PEDV strains, they have shown limited efficacy against newly emerging variants. Moreover, variant strains exhibit varying degrees of genomic mutations compared to classical strains, and the regulatory effects of these mutations on viral biology have not been systematically studied. In this study, we identify the 894–993 amino acid (aa) region within the S2 subunit as a key determinant of humoral immune evasion in variants. Mutations in this region were shown to reduce neutralization sensitivity, alter membrane fusion activity, and increase the structural rigidity of the S protein. These findings greatly enhance our understanding of the biological characteristics of PEDV variants and provide a new potential strategy and important theoretical support for viral control and vaccine development.

## INTRODUCTION

Porcine epidemic diarrhea (PED) is an acute, highly contagious intestinal infectious disease in pigs caused by porcine epidemic diarrhea virus (PEDV), leading to high mortality rates (1, 2). PED was first reported in the United Kingdom in 1971 and subsequently spread to other European countries. In 1978, the PEDV CV777 strain was first isolated and found to cause severe diarrhea in piglets while having little effect on adult pigs (3, 4). After 2010, the incidence of PED increased significantly in Asia and the Americas. China first identified the emergence of highly virulent PEDV variants (5, 6). Subsequently, outbreaks of PED in many countries (6–8) and PEDV variants caused the deaths of millions of piglets within the 1-year epidemic period (9, 10), resulting in considerable economic losses to the global swine industry.

PEDV is an enveloped, single-stranded positive-sense RNA virus encoding four structural proteins, spike (S), membrane (M), envelope (E), nucleocapsid (N), and sixteen nonstructural proteins (nsp1-16, encoded by ORF1a and ORF1b) and one accessory protein ORF3 (11, 12). Based on phylogenetic analyses of the whole genome and S gene, PEDV can be classified into two distinct genogroups: the classical genotype (GI) and the variant genotype (GII) (13). Studies have shown that since 2010, nearly all PED outbreaks have been caused by PEDV variant strains (14, 15), and herds have remained susceptible to variant strains after immunization with vaccines targeting the classical strain (CV777) (10, 16). *In vivo* assays demonstrated that immunization with an inactivated GI vaccine provided limited cross-protection against heterologous GII viruses (17). These findings suggest that vaccines based on classical PEDV strains offer poor protection against variant strains; however, the underlying mechanism is unclear.

The S protein of coronaviruses is a type I transmembrane protein that plays a crucial role in viral attachment, entry into host cells, and fusion of the viral membrane with the host cell membrane (18–21). It consists of two major domains: the S1 domain, which is responsible for receptor recognition and binding, and the S2 domain, which mediates membrane fusion. Current studies suggest that the extracellular domain of the coronavirus S protein contains multiple neutralizing epitopes and serves as a key target for humoral immune responses (22, 23).

Mutations in the SARS-CoV-2 S protein can alter antibody binding affinity, leading to immune evasion and weakening vaccine-induced immunity (23–25). Similarly, compared with PEDV GI strains, GII strains exhibit varying degrees of genomic mutations, with the S gene displaying the most significant variations (26), particularly at the N-terminus (13, 27). Although S proteins of both genotypes can induce neutralizing antibodies, cross-neutralization assays reveal significant differences (28). These findings suggest that PEDV variants may evade immunity induced by classical strain-based vaccines through alterations in key antigenic epitopes. Therefore, elucidating the molecular mechanisms driving this immune evasion is crucial for the development of effective vaccines and antiviral therapeutics.

By rescuing a series of recombinant viruses, this study identified a critical region of 894–993 amino acids in the S2 subunit as essential for immune evasion by variants that neutralize antibodies induced by classical strains. Animal challenge and immune protection experiments demonstrated that the 894–993 aa region contributed to both enhanced virulence and humoral immune evasion. Mechanistically, variant strains can evade antibody-mediated neutralization during the early stages of entry and subsequently resist neutralization through enhanced cell-to-cell transmission.

## RESULTS

### PEDV GII variant strains significantly evade the neutralizing effect of antibodies induced by classical GI strains

**Fig 1:**
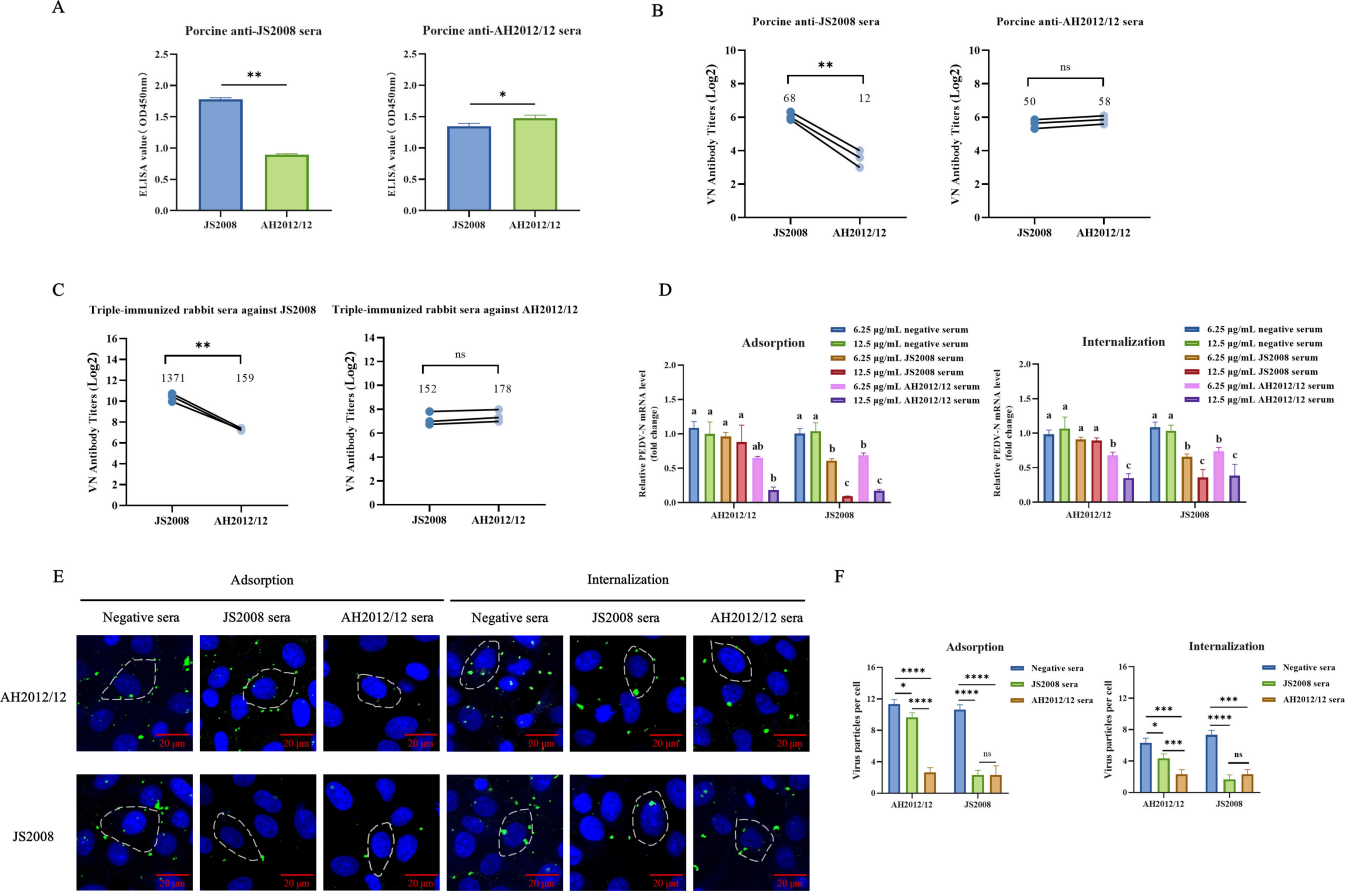
PEDV GII variant strains evade the neutralizing effect of antibodies from the classical GI strains. (**A**) Antibody titer against the virus in pig-derived hyperimmune sera. (**B**) Cross-neutralization titer of pig-derived hyperimmune sera. (**C**) Cross-neutralization titer of rabbit-derived hyperimmune sera. (**D**) The NT80 of hyperimmune sera against homologous and heterologous strains. (**E**) The purified sera were serially diluted, incubated with the virus, followed by infection of cells for 24 h. The infection was detected by IFA. NT80 was defined as the highest serum dilution that reduced viral infectivity by 80% and was used as the working concentration in the subsequent study. (**F**) Effect of hyperimmune sera on viral adsorption and internalization of homologous and heterologous strains. The data are presented as the mean ± standard deviations (SD). Statistics: Student’s *t*-test (**A–C**) or one-way ANOVA with multiple-comparison test, followed by multiple comparisons between groups (**D and F**). ns, no significance; *, *P* < 0.05; **, *P* < 0.01; ***, *P* < 0.001; and ****, *P* < 0.0001; *n* = 3. The error bars represent the standard deviations.

To investigate whether emerging PEDV variants can evade the neutralizing effect of hyperimmune sera raised against classical PEDV strains, we performed assays using porcine hyperimmune sera generated in our laboratory. As shown in Fig. 1A, porcine sera generated from the JS2008 strain (GI genotype) presented significantly reduced antibody titers against the heterologous AH2012/12 strain (GII genotype). However, sera prepared from the AH2012/12 strain showed similar titers against both the homologous (AH2012/12) and heterologous (JS2008) strains. Cross-neutralization assays revealed that the neutralization titer against the parental strain of anti-JS2008 sera was approximately 1∶68, whereas the neutralization potency against AH2012/12 was significantly reduced to 1∶12 (Fig. 1B). In contrast, anti-AH2012/12 sera with high neutralization potency against AH2012/12 still maintained relatively high neutralizing activity against JS2008 (Fig. 1B).

Subsequently, we prepared rabbit hyperimmune sera. As shown in Fig. 1C, the cross-neutralization titers of the third immunization sera prepared by the JS2008 strain were also significantly lower against AH2012/12 than against JS2008 (*P* < 0.001), with a reduction of approximately 7.2-fold. In contrast, the third immunization sera prepared from the AH2012/12 strain exhibited similar neutralization effects against both AH2012/12 and JS2008. Further analysis revealed that the anti-AH2012/12 sera effectively inhibited both homologous and heterologous strains at the adsorption and internalization stages, whereas anti-JS2008 sera exhibited inhibitory activity exclusively against the homologous strain during these two stages, with no effect observed on the heterologous strain (Fig. 1D through F). Moreover, the inhibitory effects of the hyperimmune sera at both stages were dose-dependent (Fig. 1D). Collectively, these findings demonstrated that the neutralization efficacy of PEDV hyperimmune sera elicited by the classical strain exhibited a significant drop against variant strains.

### The C terminus of S1 and the S2 subunit play critical roles in the humoral immune evasion of variant strains

**Fig 2:**
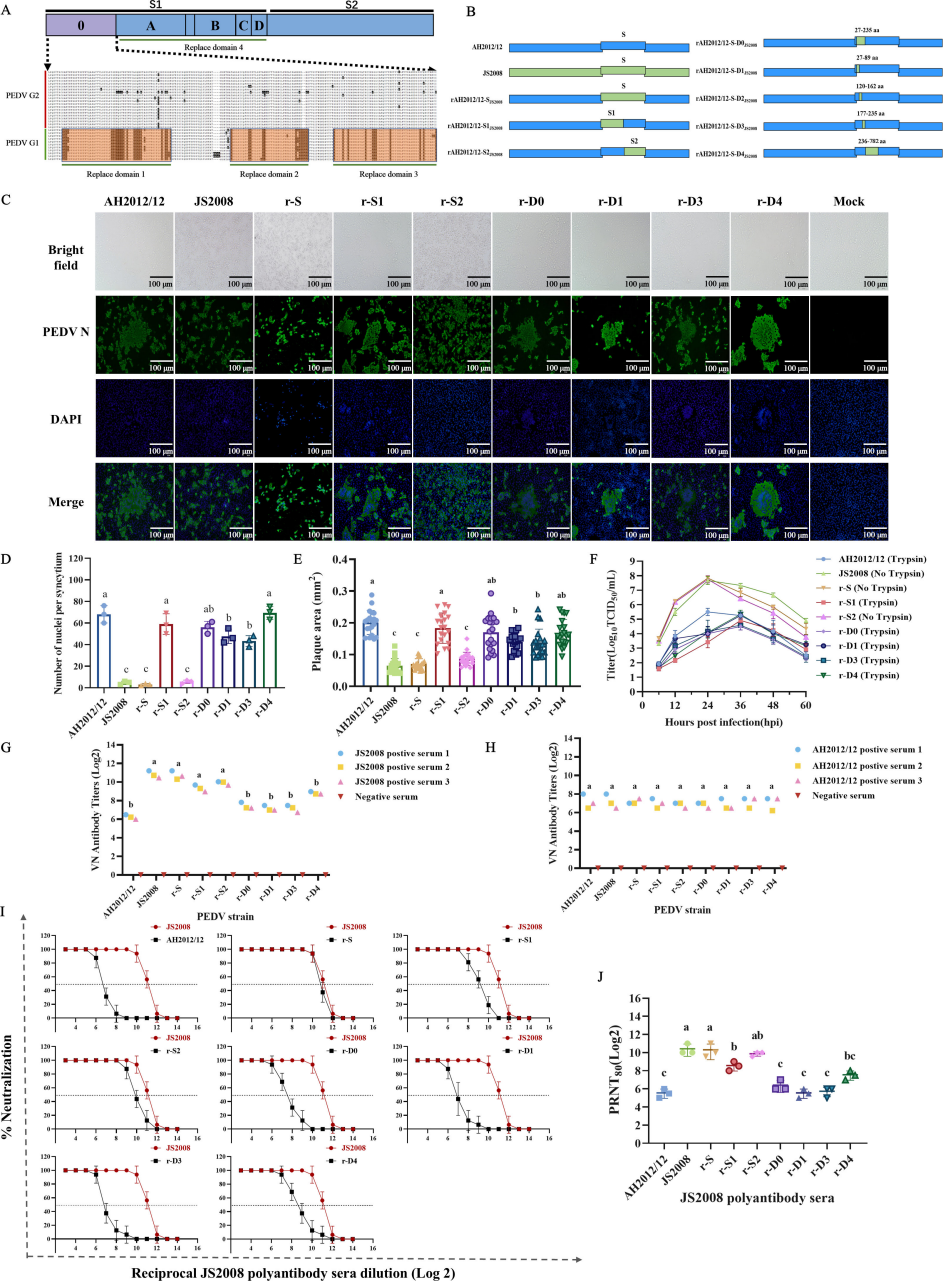
The C-termini of S1 and S2 play critical roles in the humoral immune evasion of variant strains. (**A**) Schematic diagram of the S protein structure and multiple sequence alignment. The S protein consists of S1 and S2 subunits, with the S1 subunit further divided into domains 0, A, B, C, and D. Multiple sequence alignment was performed on domain 0 (D0). The regions of differential amino acids shared by GI are highlighted in orange. These significantly variable regions were defined as replacement regions 1, 2, and 3 (D1, D2, and D3, respectively). (**B**) Strategy for constructing recombinant PEDV with large S gene segment substitutions. (**C**) CPE and IFA of parental strains AH2012/12 and JS2008, along with recombinant viruses with large-segment substitutions in the S gene. Scale bar, 100 µm. (**D**) Quantification of cell nuclei in syncytia formed by different recombinant viruses. (**E**) Statistical analysis of plaque size comparisons among parental and recombinant viruses (*n* = 20). (**F**) Growth curves of the parental strains AH2012/12 and JS2008 and recombinant viruses with large-fragment replacement in the S gene. (**G and H**) Cross-neutralization titers of rabbit-derived immune sera against AH2012, JS2008, r-S, r-S1, r-S2, r-D0, r-D1, r-D3, and r-D4. (**I**) Neutralization dose-response curves of hyperimmune anti-JS2008 sera against each strain. (**J**) Statistical analysis of the PRNT_80_ results. Statistics: one-way ANOVA with multiple-comparison test, followed by multiple comparisons between groups (**D, E, G, H, and J**). Letter labeling was used to indicate differences between groups: different letters indicate significant differences (*P* < 0.05), and the same letter indicates no significant difference (*P* > 0.05).

To investigate the key genetic regions responsible for the immune evasion of PEDV GII strains based on the functional domains of the PEDV S protein, we designed a strategy for constructing recombinant viruses with replacements in the S gene, including the full-length S, S1 subunit, S2 subunit, and D0 region. In addition, the D0, which is the most divergent region between the PEDV GI and GII strains (16, 28), was further divided into three replacement domains: 1, 2, and 3 (Fig. 2A and B). Using the CRISPR/Cas9 system, we successfully generated eight recombinant plasmids. Seven recombinant viruses were subsequently successfully rescued and identified by sequencing (Fig. S1A). The IFA results revealed that JS2008, rAH2012/12-S_JS2008_ (r-S), and rAH2012/12-S2_JS2008_ (r-S2) caused cell contraction and lysis, whereas the other five recombinant viruses, rAH2012/12-S1_JS2008_ (r-S1), rAH2012/12-D0_JS2008_ (r-D0), rAH2012/12-D1_JS2008_ (r-D1), rAH2012/12-D3_JS2008_ (r-D3), and rAH2012/12-D4_JS2008_ (r-D4), presented characteristics similar to those of the AH2012/12 strain, causing cell rounding and syncytium formation (Fig. 2C and D). Plaque assays revealed that all the strains formed nearly round plaques, but there were significant differences in plaque sizes; in particular, the trypsin-independent strains (JS2008, r-S, and r-S2) formed smaller plaques (Fig. 2E; Fig. S1B). Growth curve analysis revealed that JS2008, r-S, and r-S2 presented significantly increased viral replication rates (Fig. 2F).

To identify the key amino acid regions involved in neutralizing antibody evasion, cross-neutralization assays were performed. As shown in Fig. 2G, the JS2008 hyperimmune sera effectively neutralized r-S2, r-S1, and r-D4. In contrast, the neutralization titers against other recombinant viruses were significantly reduced. The hyperimmune sera anti-AH2012/12, however, exhibited high neutralization titers against all strains (Fig. 2H). The neutralization dose-response curves and plaque reduction neutralization test (PRNT) also revealed that the PEDV S gene plays a vital role in evading neutralizing antibodies from classical strains, especially the amino acid mutations in the C-terminus of S1 (D4) region and S2 subunit (Fig. 2I and J; Fig. S2A).

### The 894–993 aa region is a critical mutation region for humoral immune evasion in variant strains

**Fig 3:**
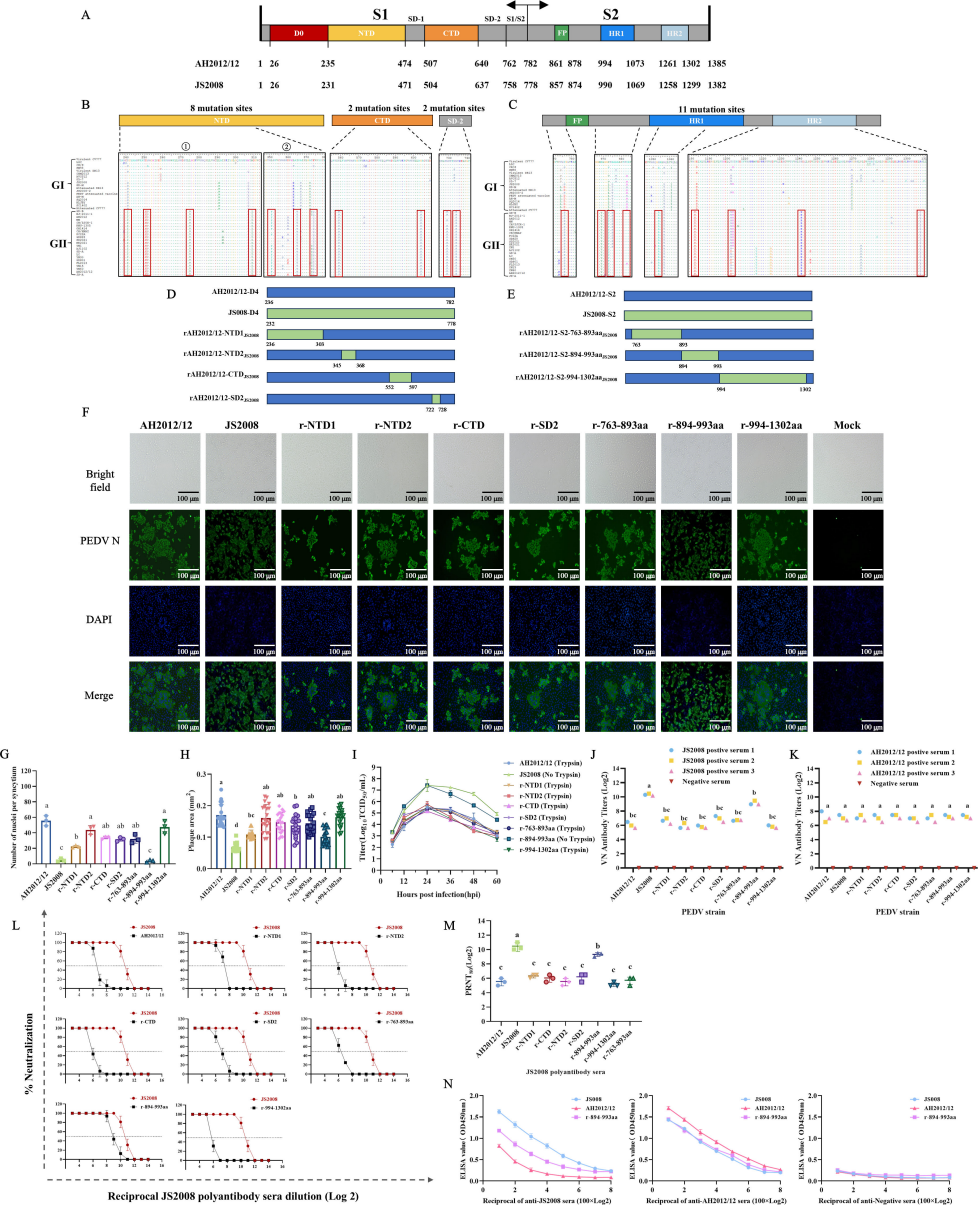
The 894–993 aa is a critical mutation region for humoral immune evasion in variant strains. (**A**) Schematic diagram of the PEDV S1 and S2 gene domains. (**B and C**) Multiple sequence alignment of the D4 region and the S2 gene. Owing to the high mutation frequency in the NTD, this region was further subdivided into NTD1 and NTD2. (**D and E**) Strategy for constructing recombinant PEDV strains with small-segment substitutions. (**F**) CPEs and IFA of parental strains and recombinant viruses with small-segment replacements in the S gene. Scale bar, 100 µm. (**G**) Quantification of cell nuclei in syncytium formed by different recombinant viruses. (**H**) Statistical analysis of plaque sizes for parental strains and recombinant viruses with small-segment replacements in the S gene (*n* = 20). Different letters indicate statistically significant differences between groups (*P* < 0.05), whereas the same letters indicate no statistically significant difference (*P* > 0.05). (**I**) Growth curves of parental strains AH2012/12 and JS2008, along with recombinant viruses with small-segment replacements in the S gene. (**J and K**) Cross-neutralization titers of rabbit-derived hyperimmune sera against AH2012, JS2008, and recombinant viruses with small-segment replacements in the S gene (r-NTD1, r-NTD2, r-CTD, r-SD2, r-763-893aa, r-894-993aa, and r-994-1302aa). (**L**) Neutralization dose-response curves of rabbit-derived anti-JS2008 sera against each strain. (**M**) Statistical analysis of the PRNT. (**N**) ELISA analysis of differential antibody-binding activities among strains. ELISA was performed by coating equal amounts of viruses (100 TCID_50_) and using serially diluted sera as the primary antibody to compare the antibody-binding activities of different strains. Statistics: one-way ANOVA with multiple-comparison test, followed by multiple comparisons between groups (**G, H, J, K, and M**). Different letters indicate statistically significant differences between groups (*P* < 0.05), whereas the same letters indicate no statistically significant difference (*P* > 0.05).

To further screen the key amino acid regions, we performed multiple sequence alignment of the D4 region and S2 subunit of the PEDV GI and GII strains and annotated high-frequency mutant amino acids in known functional domains (Fig. 3A through C). Based on these comparisons, we designed a strategy for constructing recombinant viruses with small-fragment replacements (Fig. 3D and E) and successfully generated recombinant plasmids (Fig. S3A). Subsequently, by transfecting the recombinant plasmids into Vero cells, we successfully rescued seven stable-replicating recombinant viruses and reported that rAH2012/12-894-993aa_JS2008_ was a trypsin-independent strain. The CPE of r-894-993aa was characterized mainly by cell lysis and no cell fusion (Fig. 3F and G). The plaques formed by JS2008, rAH2012/12-N-terminal domain 1 (r-NTD1), and r-894-993aa were smaller (Fig. 3H; Fig. S3B). The virus growth results revealed that r-894-993aa exhibited growth kinetics similar to those of JS2008 and maintained significantly higher viral titers than the other viruses within 60 h post-infection (hpi) (Fig. 3I). In contrast, the proliferation efficiencies of the other strains were similar to those of AH2012/12.

A series of cross-neutralization assays revealed that anti-JS2008 sera strongly neutralized the recombinant virus r-894-993aa and had a mild neutralizing effect on r-NTD1 and rAH2012/12-subdomain 2 (r-SD2) (Fig. 3J). Additionally, consistent with previous findings, the antisera against AH2012/12 displayed robust neutralization activity against all the tested strains (Fig. 3K). On this basis, as shown in Fig. 3L, the neutralization curve of the r-894-993aa strain was closest to that of JS2008, indicating a greater sensitivity to anti-JS2008 sera than to other recombinant strains. PRNT also revealed that, compared with AH2012/12, r-894-993aa exhibited significantly increased sensitivity to anti-JS2008 sera (Fig. 3M; Fig. S4A). Additionally, enzyme-linked immunosorbent assay (ELISA) assays further confirmed that the JS2008 parental virus and r-894-993aa exhibited strong binding activity with anti-JS2008 sera antibodies, whereas the AH2012/12 virus demonstrated poor binding capacity with these antibodies (Fig. 3N). These results confirmed that mutations within the 894–993 aa segment play a critical role in enabling variant strains to evade neutralization by classical strain antibodies.

### Substitution of the 894–993 aa region reduces the pathogenicity of PEDV variants

**Fig 4:**
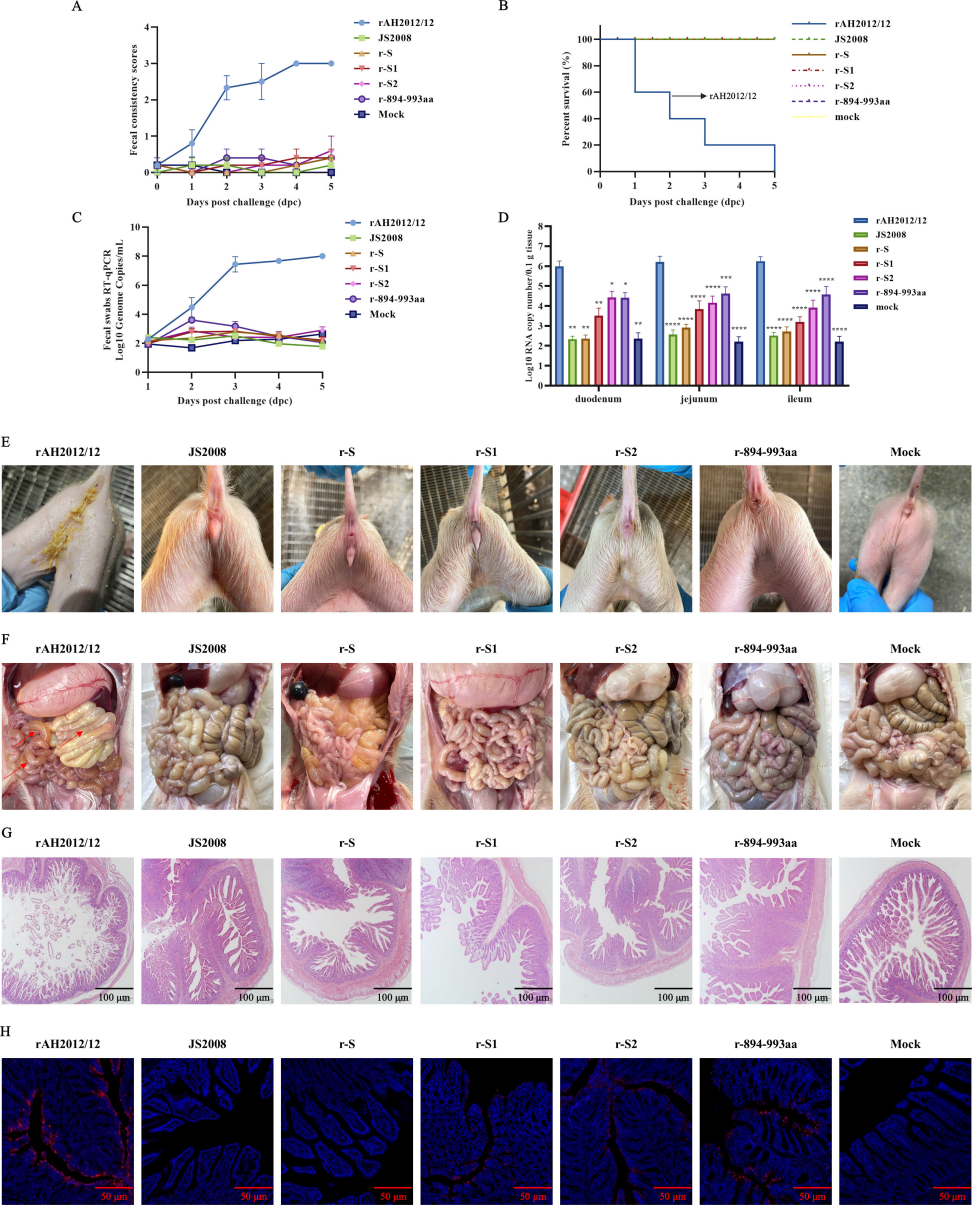
Substitution of the 894–993 aa region reduces the pathogenicity of PEDV variants. (**A**) Diarrhea scores of challenged piglets. (**B**) Survival rate of challenged piglets. (**C**) Daily viral shedding in rectal swabs from challenged piglets. (**D**) Viral loads in the duodenum, jejunum, and ileum of challenged piglets at 5 dpc. Clinical diarrhea conditions (**E**), gross lesion observation (**F**), histopathological examination (**G**), and IFA (**H**) of intestinal tissues from challenged piglets. The PEDV N protein was labeled with red fluorescence, and the cell nuclei were stained with DAPI. Scale bar, 100 µm. Data were analyzed by one-way ANOVA with post hoc comparisons compared with the rAH2012/12-challenged group (**D**). *P < 0.05, **P < 0.01, ***P < 0.001, and ****P < 0.0001.

PEDV GI strains, such as CV777, DR13, and JS2008, generally exhibit lower virulence, whereas PEDV GII strains demonstrate greater pathogenicity (17). To further evaluate the virulence of the recombinant viruses, piglets were orally challenged with rAH2012/12, JS2008, r-S, r-S1, r-S2, and r-894-993aa. In the rAH2012/12 challenge group, watery diarrhea appeared at 1 day post-challenge (dpc), with further worsening at 4–5 dpc. In contrast, no significant clinical signs were observed in the other groups or the blank group (Fig. 4A and E). Mortality occurred in only the AH2012/12 challenge group, followed by two deaths at 1 dpc and one death each at 2, 3, and 5 dpc (Fig. 4B).

Rectal swab analysis revealed viral shedding in the rAH2012/12-infected group beginning at 2 dpc, with viral loads increasing progressively from 2 to 5 dpc, reaching 10^8^ copies/mL (Fig. 4C). In contrast, the r-894-993aa-challenged group exhibited only low-level viral shedding at 2–3 dpc, and no significant PEDV RNAs were detected in the other challenge groups. Necropsy of the rAH2012/12-infected piglets revealed distended, thinned small intestines filled with gas and water. In contrast, intestines from other challenge groups showed no obvious thinning or hemorrhaging (Fig. 4F). High levels of viral RNA (10^6^ copies/0.1g) were detected in the duodenum, jejunum, and ileum of the rAH2012/12-infected group. Still, they were significantly lower in the r-894-993aa-infected group (Fig. 4D).

Histopathological analysis revealed severe villous atrophy, fragmentation, and necrotic debris in the ileum of the rAH2012/12-infected piglets; mild villous atrophy in the r-894-993aa-challenged group; and intact leaf-like villi in the other groups and the control group (Fig. 4G). Figure 4H shows substantial positive signals in the rAH2012/12 group, with moderate signals in the r-894-993aa group, while the other groups show weak signals. These data suggest that both r-S1 and r-S2 exhibit significantly reduced virulence compared with AH2012/12, indicating that both subunits contribute to virulence, with the 894–993 aa region within the S2 being essential for enhanced virulence in GII strains.

### *In vivo* experiments confirmed that mutations in the 894–993 aa region enable variants to evade neutralizing antibodies from classical strains

An *in vivo* immunization-challenge experiment was performed to further validate the critical role of the 894–993 aa region in mediating the immune evasion of GII strains from GI strain-induced protection, with the rAH2012/12 and r-894-993aa inactivated vaccines prepared. First, vaccine immunogenicity was assessed in a mouse model (Fig. S5A). The results revealed no significant difference in the serum antibody titers between the mice immunized with rAH2012/12 and those immunized with r-894-993aa (Fig. S5B). Subsequently, cross-neutralization assays were conducted using high-titer mouse sera against r-894-993aa. As shown in Fig. S5C, these sera exhibited the greatest neutralizing effect against r-894-993aa, a slightly lower neutralization titer against JS2008, and a significantly reduced titer against AH2012/12.

**Fig 5:**
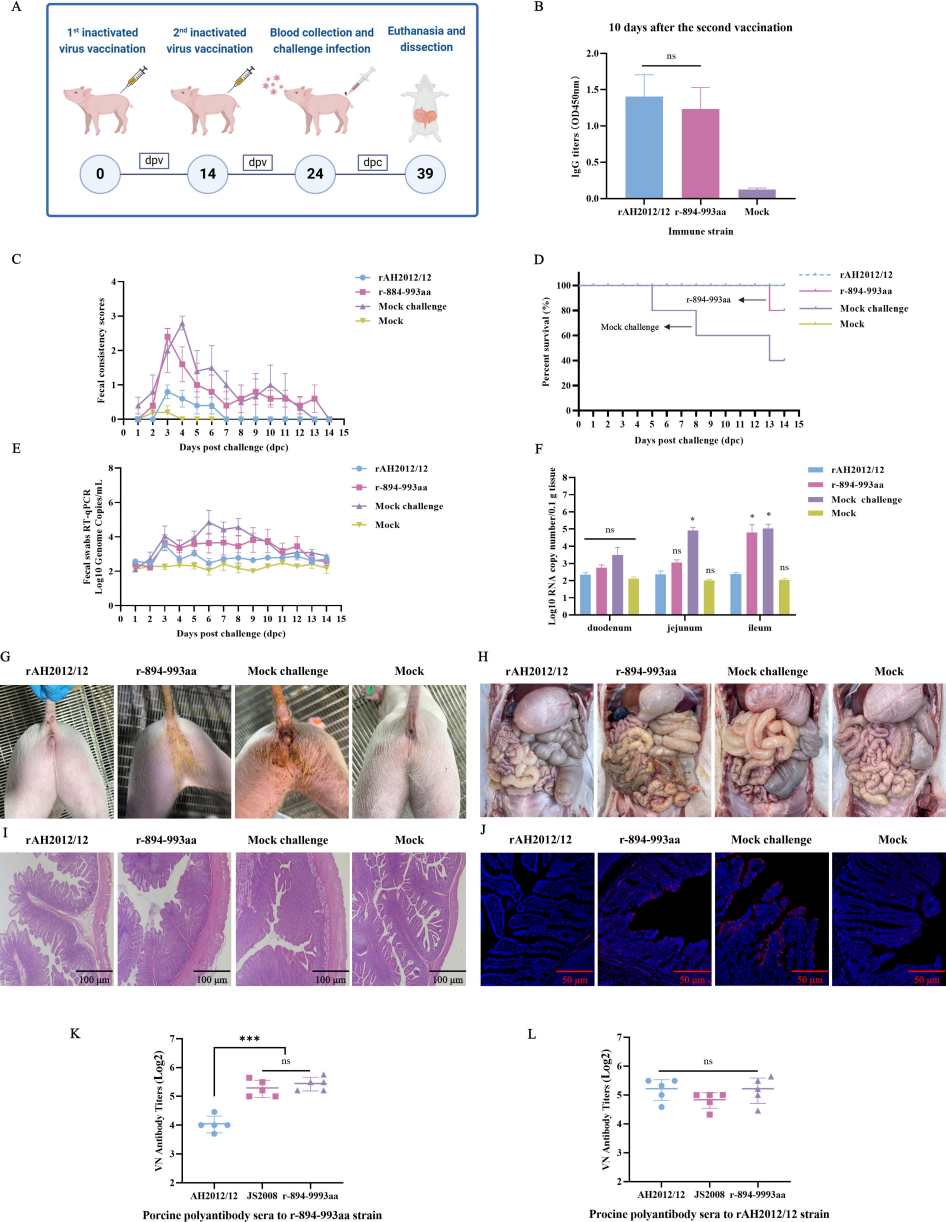
*In vivo* experiments confirmed that mutations in the 894–993 aa region enable variants to evade neutralizing antibodies from classical strains. (**A**) Immunization and challenge assay design for rAH2012/12 and r-894-993aa. (**B**) Antibody titers in immune sera. Sera were collected 10 days after the second immunization from each group, and total IgG antibody titers against the PEDV S1 protein were measured. (**C**) Diarrhea scores of challenged piglets. (**D**) The survival rate of the challenged piglets in each group. (**E**) Daily viral shedding in rectal swabs from challenged piglets. (**F**) Viral loads in the duodenum, jejunum, and ileum of challenged piglets at 5 dpc. (**G**) Gross lesion observation (**H**), histopathological examination (**I**), and IFA (**J**) of intestinal tissues in challenged piglets. Scale bar, 100 µm. (**K and L**) Cross-neutralization results of porcine immune sera against rAH2012/12 and r-894-993aa. Statistics: Differences among groups were analyzed by one-way ANOVA followed by multiple-comparison tests (**B, K, and L**); differences between each experimental group and the rAH2012/12 group were analyzed by one-way ANOVA with multiple comparisons (**F**). Statistical significance is indicated as follows: ns, no significance; *P < 0.05, **P < 0.01, ***P < 0.001, and ****P < 0.0001.

We further evaluated the immunogenicity and cross-protective efficacy of the parental and recombinant strains in piglets, as outlined in Fig. 5A. Sera IgG levels at 10 days after the second immunization showed that both the rAH2012/12 and the r-894-993aa vaccines induced high levels of antibodies in the piglets, with no significant difference between the immunized groups (Fig. 5B). Subsequently, groups 1–3 were challenged orally with 2 × 10^6^ TCID_50_/mL of rAH2012/12 and group 4 was given DMEM as a control. Clinical symptom monitoring revealed that both the r-894-993aa-immunized challenge group and the non-immunized challenge group exhibited similar diarrheal progression, with severe diarrhea occurring at 2–3 and 3–4 dpc, respectively, followed by alleviation, although moderate diarrhea persisted until 13 dpc (Fig. 5C and G). However, in the rAH2012/12-immunized challenge group, mild diarrhea was observed at 3–6 dpc, after which the diarrhea symptoms disappeared (Fig. 5C and G). Survival rate analysis (Fig. 5D) revealed that three piglets died at 5, 8, and 13 dpc in the non-immunized challenge group, and one death at 13 dpc in the r-894-993aa group, whereas no mortality occurred in the rAH2012/12-immunized challenge group and the control group. As shown in Fig. 5E, viral shedding in the non-immunized challenge group was first detected at 3 dpc, with high levels of approximately 10^4.8^ copies/mL and continued throughout the study period. The r-894-993aa-immunized challenge group presented with virus shedding at 3 dpc, which was consistently detected from 4 to 12 dpc, with a peak at 10^3.6^ copies/mL. Only low levels of viral RNA were detected in the rAH2012/12-immunized challenge group, while almost no viral RNA was detected in the control group.

Analysis of viral loads revealed that both the r-894-993aa-immunized challenged group and the non-immunized challenged group presented high viral loads in the ileum, reaching approximately 10⁵ copies/mL, whereas the rAH2012/12-immunized group presented levels comparable to those of the negative control group (Fig. 5F). Gross examination revealed severe intestinal thinning and distension in the non-immunized challenge group, mild lesions in the r-894-993aa group (Fig. 5H). Histological analysis revealed severe villous atrophy in the non-immunized challenge group, moderate villous shortening in the r-894-993aa-immunized challenge group, and intact villi in the other two groups (Fig. 5I). IFA revealed many PEDV N protein-specific fluorescence signals in the epithelial cells of the villi in the non-immunized challenge group, reduced signals at the villus tips in the r-894-993aa-immunized challenge group, and minimal signals in the rAH2012/12-immunized challenge group (Fig. 5J). Furthermore, the results of cross-neutralization assays revealed that anti-r-894-993aa sera exhibited better neutralization efficacy against r-894-993aa and JS2008, but poorly neutralized AH2012/12 (Fig. 5K). In contrast, high-titer anti-AH2012/12 sera effectively neutralized multiple viruses (Fig. 5L). These findings confirmed that the 894–993 aa region contains critical neutralizing epitopes and that its mutations enable variant strains to evade neutralizing antibodies induced by classical strains.

### Mutations in the 894–993 aa region increase cell-to-cell transmission and confer serum neutralization resistance to variants

**Fig 6:**
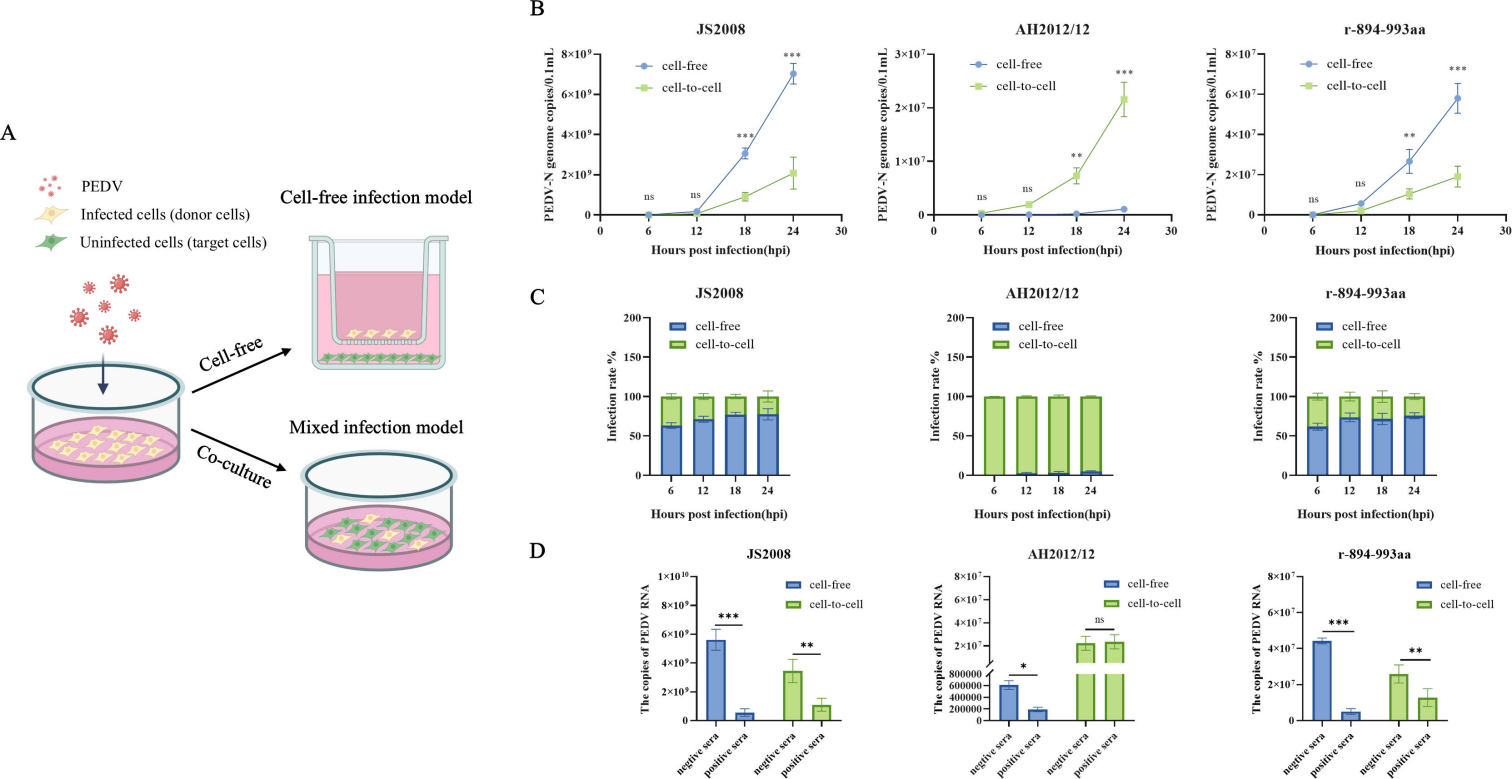
Mutations in the 894–993 aa region enhance cell-to-cell transmission and confer sera neutralization resistance to variants. (**A**) Schematic diagram of the cell-to-cell and cell-free transmission models. (**B**) Viral replication kinetics of the JS2008, AH2012/12, and r-894-993aa strains. (**C**) Comparison of the infection rates of JS2008, AH2012/12, and r-894-993aa under cell-to-cell and cell-free transmission conditions. (**D**) Effects of JS2008-positive and negative sera on the cell-to-cell and cell-free infection of different strains. Statistics: Student’s *t*-test (**B and D**). Statistical significance is indicated as follows: ns, no significance; *P < 0.05, **P < 0.01, ***P < 0.001, and ****P < 0.0001.

Given the loss of cell fusion activity in the recombinant virus r-894-993aa, we prompted an investigation into its transmission mode. Using Transwell inserts and coculture systems, we established cell-free and cell-to-cell transmission models for JS2008, AH2012/12, and r-894-993aa (Fig. 6A). Infection kinetics revealed that JS2008 primarily relied on cell-free transmission, whereas AH2012/12 predominantly spread via cell-to-cell contact. Interestingly, r-894-993aa displayed a transmission profile similar to that of JS2008, establishing rapid infection among target cells through cell-free transmission (Fig. 6B). We then compared the infection efficiency of the three strains at various time points. As shown in Fig. 6C, JS2008 exhibited a significantly higher rate of cell-free infection, ranging from 63% to 77%, compared to cell-to-cell spread. In contrast, AH2012/12 demonstrated extremely high efficiency of cell-to-cell transmission, reaching nearly 95%, with minimal contribution from cell-free spread. The transmission mode of r-894-993aa was time-dependent, with approximately 76% of infections via the cell-free spread and 24% via cell-to-cell spread at 24 hpi. These findings indicate that different subtypes of PEDV adopt distinct transmission strategies, and that the 894–993 aa region of the S gene plays a critical role in determining the transmission mode.

Based on these findings, we assessed the sensitivity of each transmission mode to neutralizing sera derived from classical strains. Anti-JS2008 sera and negative control sera were purified via protein A＋G affinity chromatography. After two infection models were established as described in Materials and Methods, the purified positive sera with a 1×NT50 concentration and negative sera with equal concentrations were added, and viral copy numbers were quantified at 24 hpi. In the JS2008-infected group, anti-JS2008 sera significantly suppressed both cell-free and cell-to-cell transmission, with a stronger inhibitory effect on cell-free spread (Fig. 6D). For AH2012/12, the sera inhibited cell-free infection but had no discernible effect on cell-to-cell spread (Fig. 6D). Notably, the responses observed in the r-894-993aa-infected group were consistent with those of JS2008, with neutralizing antibodies effectively reducing viral replication via both transmission routes (Fig. 6D). These data revealed that mutations in 894–993 aa of variant strains enhance cell-to-cell transmission and reduce neutralizing sensitivity to high immune sera induced by classical strains.

### Mutations in the 894–993 aa region alter the structure and characteristics of the S protein in variants

**Fig 7:**
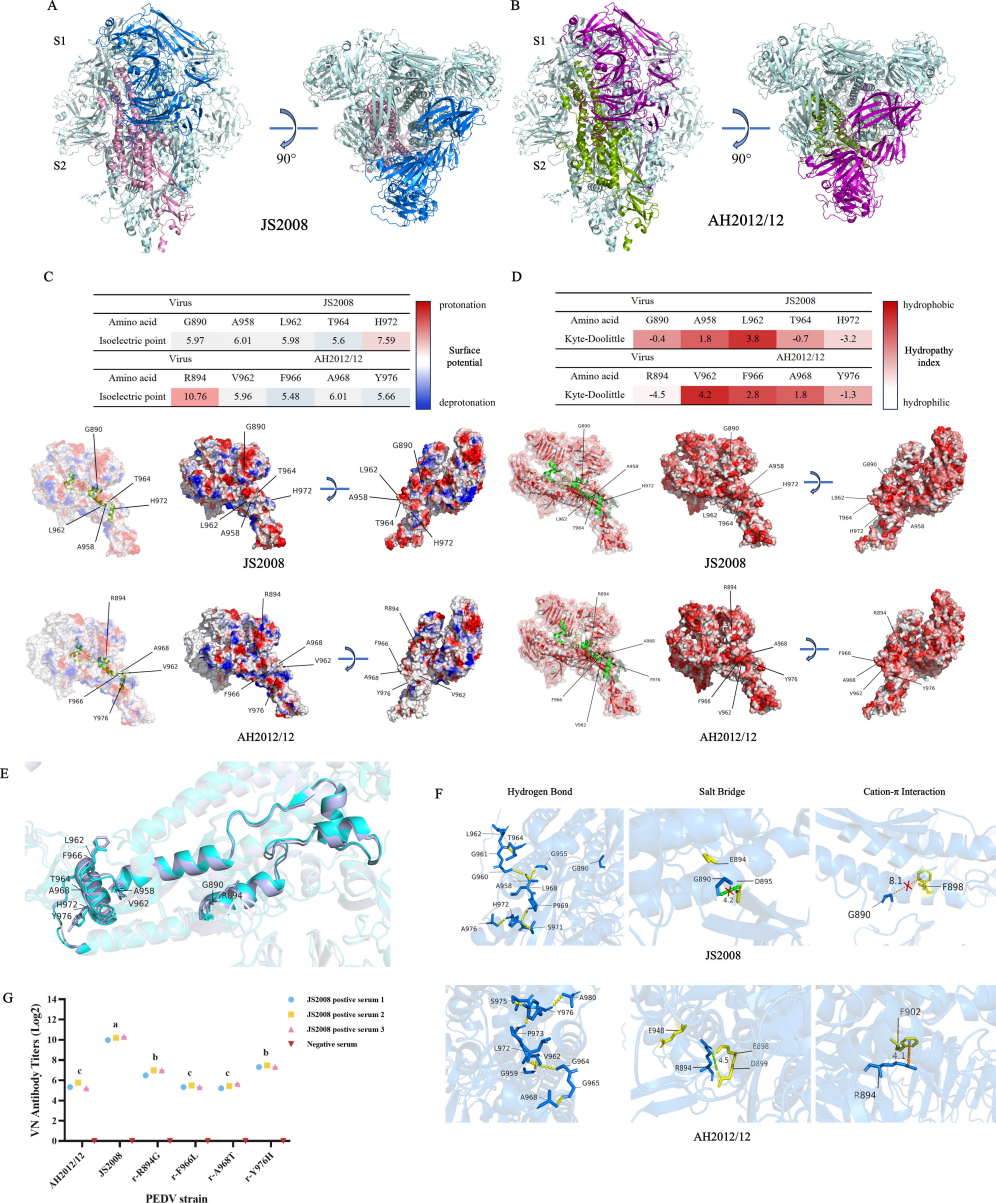
Mutations in the 894–993 aa region alter the structure and characteristics of the S protein in variants. (**A and B**) Homology modeling of the S protein trimers of JS2008 and AH2012/12. The trimeric backbones are shown in cartoon cyan. For JS2008, the S1 and S2 subunits are colored in blue and pink, respectively. For AH2012/12, the S1 and S2 subunits are shown in purple and green, respectively. Both front and top views of the trimers are displayed. (**C**) Electrostatic surface potential distribution of the 890–989 aa region in JS2008 and the equivalent region of 894–993 aa in AH2012/12. (**D**) Hydrophobicity of the corresponding 890–989 aa region in JS2008 and the 894–993 aa region in AH2012/12. The 3D structures highlighted the spatially folded conformation of these regions using green cartoon models, with each residue labeled by a single letter and its corresponding sequence position. (**E**) Comparison of the secondary structures in the equivalent regions of JS2008 and AH2012/12. The AH2012/12 sequence is represented in purple, and the JS2008 sequence is represented in cyan. (**F**) Intramolecular interaction analysis of the 890–989 aa region of JS2008 and the 894–993 aa equivalent region of AH2012/12, including hydrogen bonds, salt bridges, and cation-π interactions. (**G**) Cross-neutralization titers of anti-JS2008 sera against single-site mutant recombinant viruses. Statistics: one-way ANOVA with multiple-comparison test, followed by multiple comparisons between groups (**G**).

The S proteins of JS2008 and AH2012/12 were homology modeled using the SWISS-MODEL, and structural reliability was assessed by GMQE and QMEAN scores. The results indicated high similarity, with a root mean square deviation (RMSD) of 0.084 Å between the models, indicating no significant differences in overall conformation (Fig. 7A and B). Comparative analysis of the 890–989 aa region in JS2008 and the equivalent 894–993 aa region in AH2012/12 revealed differences in surface potential and hydrophobicity. Five amino acid mutations are shown in Fig. 7C, in which G890, A958, L962, T964, and H972 of JS2008 were replaced by R894, V962, F966, A968, and Y976 of AH2012/12. The surface potential of amino acids is mainly determined by the acid-base properties of the side chains and the ionization state. Based on the isoelectric points of the amino acids, we generated a heatmap to visualize the electrostatic potential under neutral conditions (pH 7.0). The analysis revealed that the G890 to R894 mutation altered the residue from neutral to positively charged, whereas the H972 to Y976 mutation led to a partial loss of positive charge. The protein structure models in Fig. 7C further illustrated the surface charge distribution, suggesting potential impacts on the solubility and stability of the S protein. In addition, the hydrophobicity of amino acids is determined primarily by the polar groups of the side chains. Multiple studies have utilized hydrophilic residues to predict epitopes, demonstrating a strong correlation between hydrophilicity peaks and identified epitopes (29, 30). As shown in Fig. 7D, the 890–989 aa region of the JS2008 strain was enriched in hydrophilic amino acids, which were typically exposed on the protein surface, having the potential to form epitopes. In contrast, the 894–993 aa region of the AH2012/12 strain presented an increased proportion of hydrophobic residues, enhancing the tendency of the S protein to fold inward, which might result in epitope masking or loss, thereby impairing antibody recognition.

We subsequently analyzed the secondary and tertiary structures of the S protein. Figure 7E presents a comparison of the secondary structures of the equivalent 890–989aa region in JS2008 and the 894-993 aa region in AH2012/12. Although no significant differences in overall protein folding were detected between the classical and mutant strains (RMSD = 0.082), changes in intermolecular interactions were detected (Fig. 7F). The computational results revealed that key amino acid residues within the equivalent regions of JS2008 and AH2012/12 were able to form similar hydrogen bonds. However, only R894 in AH2012/12 carried a positive charge, and its amino group formed a salt bridge with the carboxyl group of D899 within a 5.5 Å range, with an intermolecular distance of 4.5 Å. In contrast, G890 in JS2008 lacks a charged side chain and thus cannot form a salt bridge with D895. Furthermore, we determined that R894 in AH2012/12 was able to form a cation-π interaction with F902 (4.1 Å), whereas in JS2008, F890 was too distant from F898 (8.1 Å) to form this interaction. Structural analyses indicate that the 894–993 aa region of the variants has physicochemical properties distinct from those of the classical strain. The variants may modulate surface electrostatic potential to alter antibody binding affinity, and the increased proportion of hydrophobic residues promotes protein folding. Additionally, the formation of the salt bridge and cation-π interaction can further stabilize the protein conformation and restrict structural flexibility.

We further generated recombinant viruses carrying single-site mutations within the 894–993 aa region. Except for V962A, the other four mutants were successfully rescued and named r-R894G, r-F966L, r-A968T, and r-Y976H (Fig. S6A). Notably, r-Y976H was able to be serially passaged in the absence of trypsin (Fig. S6B). The growth curves showed that only r-Y976H exhibited a replication kinetics level closer to that of JS2008, although it remained significantly lower than the parental JS2008 strain (Fig. S6C). Plaque assays showed that, except for r-F966L, the plaques formed by r-R894G, r-A968T, and r-Y976H were all smaller than the parental AH2012/12 strain (Fig. S6D). These results suggest that multiple sites in this region determine the dynamics of viral replication. In addition, cross-neutralization assays revealed a slight increase in neutralizing activity of anti-JS2008 sera against r-R894G and r-Y976H, indicating that these substitutions can contribute to humoral immune evasion (Fig. 7G). Collectively, these results indicate the 894–993 aa region collectively determines the viral biological properties, and the mutations at residues 894 and 976 contribute more prominently to immune evasion.

## DISCUSSION

The large differences in phylogeny and antigenic epitopes between PEDV classical and field-prevalent variant strains allow the variant strains to partially or completely evade the humoral immune response induced by classical strains (31, 32).

Previous studies have suggested that the neutralizing epitopes of the PEDV S protein are predominantly localized in the S1 subunit, including multiple structural domains (regions 0 and A–D) (33, 34) and the collagenase equivalent (COE) region (amino acid residues 499–638) (35). However, emerging evidence has demonstrated that the S2 subunit possesses significant immunogenicity comparable to that of S1, with critical neutralizing epitopes identified in its N-terminal (aa 744–771) and C-terminal (aa 1371–1,377) regions (23, 36). In this study, we further revealed that the NTD and SD2 of the S1 subunit were partially associated with immune evasion. Most importantly, we first identified and validated that the 894–993 aa region within the S2 subunit plays a key role in the immune evasion of PEDV variants. This region has not been recognized as a neutralizing epitope and was not associated with immune evasion in earlier studies. Further multisequence alignment revealed that mutations within the 894–993 aa region were highly conserved among GII subtype strains, suggesting that amino acid changes might represent an adaptive evolution of PEDV under immune pressure imposed by GI vaccines.

The PEDV S2 subunit plays a critical role in regulating trypsin dependence and influences virus-induced cell fusion (37, 38). However, the key amino acid region responsible for modulating trypsin dependence is still unclear. In this study, by constructing a series of recombinant viruses with S2 subunit substitutions, we identified the 894–993 aa as the essential region regulating both the trypsin dependence and cell fusion activity of PEDV for the first time. Membrane fusion-dependent transmission is the primary route for the spread of coronaviruses. SARS-CoV-2 and PDCoV have been shown to utilize this mode of transmission to evade neutralizing antibodies (39, 40). Recent research on FCoV-23 further revealed that loss of the D0 domain enhances membrane fusion and accelerates host cell entry (41). In our study, we observed that r-894-993aa lost cell fusion ability, indicating that the 894–993 aa region of the PEDV S protein determines the membrane fusion capability of the strain. We further systematically analyzed the transmission modes of PEDV subtypes and the contributions of these modes to neutralizing antibody evasion. The results demonstrated that the AH2012/12 strain was able to resist neutralizing antibodies via cell-to-cell spread, whereas the JS2008 strain and r-894-993aa, which primarily utilized cell-free spread, were strongly inhibited by neutralizing sera. In addition, we confirmed that hyperimmune sera induced by the classical strain effectively inhibited homologous virus infection at both the adsorption and internalization stages; however, this effect was lost against heterologous strains at the same stages. This study provides direct evidence that PEDV variants are capable of evading neutralizing antibodies during the early stages of viral invasion. Notably, after successful infection of host cells, the variants can further evade antibody-mediated neutralization by enhancing cell-to-cell spread through membrane fusion. These findings reveal that PEDV variants employ a dual immune evasion strategy, comprising early-phase evasion and efficient intercellular transmission to evade host humoral immunity.

Several studies confirmed the pathogenic domains in the S gene, including deletions in the NTD (42), aa 62 in D0 and aa 722 in SD2 of the S1 subunit (43), substitutions in the S2 subunit (44), the KVHVQ motif at the cytoplasmic tail (45), and a 7-aa deletion at the end of the S2 subunit (46, 47). In this study, we demonstrated that substituting the S gene, S1, S2, or even the 890–989 aa region from the JS2008 strain into the AH2012/12 backbone significantly attenuated viral virulence. These findings confirmed that the S gene is a virulence determinant and identified multiple regions within it as key regulatory elements involved in modulating pathogenicity. Recent studies have shown that trypsin activity is high in the pancreas of neonatal piglets and increases during the weaning period (48). In addition, Wicht et al. (49) previously demonstrated that the S protein of the trypsin-independent strain PEDV-Sca was cleaved by trypsin, resulting in a marked reduction in infectivity. In this study, we observed a low viral load in the intestines of piglets infected with r-894-993aa, along with a notable decrease in mortality. Considering the low fusion activity and trypsin independence of the recombinant virus *in vitro*, we speculate that r-894-993aa may be similar to PEDV-Sca, with reduced resistance to trypsin. Therefore, during natural infection, r-894-993aa is exposed to high levels of trypsin in the intestines, leading to cleavage of the S protein and reduced viral infectivity, resulting in significant attenuation *in vivo*.

In the reverse genetic system, it is a relatively common phenomenon that some recombinant viruses fail to rescue, which can be attributed to the instability of some viral sequences in bacteria and to extensive amino acid mutations that may disrupt protein folding, processing, or interactions of the protein, thereby impairing efficient virion assembly (50). In this study, the virus that could not be rescued involved replacing 120–162 aa of AH2012/12, which contains multiple nonsynonymous substitutions and introduces an N-glycosylation site, possibly altering local conformation and interfering with S protein folding or virion assembly. Meanwhile, r-S and r-S2 exhibited significantly higher replication levels at 6 hpi, consistent with previous reports implicating the S protein in regulating PEDV replication kinetics (38, 51). We further observed that trypsin-independent strains exhibited higher replication efficiency than trypsin-dependent strains, which we speculate is due to the S protein enhancing viral assembly and egress efficiency.

The S2 subunit of coronaviruses mediates membrane fusion through conformational changes, and the connecting region (CR) between the fusion peptide (FP) and the heptad repeat 1 (HR1) is considered critical for maintaining structural stability and fusion efficiency  (52). Previous studies have shown that L898 and N901 mutations within the CR and HR1 regions of the SARS-CoV S protein disrupt the stability of the HR1 α-helix and significantly reduce the cell fusion capacity mediated by the S protein (53). In this study, the identified 894–993 aa segment corresponds to the CR domain of the PEDV S protein. Therefore, mutations in the CR may impair forward helical structure formation by destabilizing the HR1 conformation and restricting FP insertion into the host membrane, thereby comprehensively weakening S protein-mediated membrane fusion (54).

In addition, specific amino acid mutations may alter the physicochemical properties of viral proteins, resulting in changes in surface potential, increased hydrophobicity, and strengthened noncovalent interactions, which are considered key factors in reducing neutralizing antibody affinity (55, 56). In this study, the R894, V962, and Y976 mutations at aa 894–993 resulted in a significant change in surface electrostatic potential and increased hydrophobicity. Additionally, the R894G mutation introduced a new salt bridge and a cation-π interaction. These mutations resulted in (i) alterations of the antigenic epitope within the 894–992 aa region of variants, impairing the binding of neutralizing antibodies elicited by the classical 890–989 aa epitope and (ii) mutations in the 894–993 aa region also formed a new critical neutralizing epitope in the variant strains. To validate the functional impact of the structural changes, we generated recombinant viruses carrying single-site mutations. The results showed that the R894G, A968T, and Y976H mutations resulted in smaller plaques, with r-Y976H exhibiting enhanced replicative capacity. In addition, anti-JS2008 sera exhibited increased neutralizing activity against r-R894G and r-Y976H, indicating that the mutations of 894–993 aa region collectively determine the viral biological properties, and the mutations at residues 894 and 976 contribute more prominently to immune evasion.

Moreover, the 894–993 aa region also provides valuable insights for vaccine design. Variants-specific epitopes within this region can guide the development of subunit or recombinant vaccines to enhance broad protection against GII strains. This region can serve as an antigenic peptide for developing neutralizing monoclonal antibodies and a potential site for antiviral drug development, thereby supporting more precise strategies for PEDV prevention and control.

In summary, this study confirms for the first time that PEDV variants can evade humoral immune responses induced by classical strains and identifies the 894–993 aa region of the S gene as a key regulatory determinant. This region plays a central role not only in regulating the predominant mode of viral transmission but also in modulating viral virulence and immune evasion. These findings expand the current understanding of functional domains within the PEDV S gene and elucidate the molecular mechanism underlying humoral immune evasion of variants, providing important theoretical insights for coronavirus vaccine development and control strategies.

## MATERIALS AND METHODS

### Cells, virus strains, and antibodies

Vero cells (ATCC no. CCL-81) were maintained in our laboratory and cultured in Dulbecco’s modified Eagle’s medium (DMEM; BasalMedia Technologies, Shanghai, China) supplemented with 10% fetal bovine serum (FBS; Vazyme Biotech, Nanjing, China). All the PEDV strains used in this study were isolated and preserved in our laboratory. The GI-type JS2008 strain (GenBank accession no. KC109141) was propagated in Vero cells with virus maintenance medium supplemented with 2% FBS. Similarly, the GII-type AH2012/12 strain (GenBank accession no. KU646831) was propagated in Vero cells, with virus maintenance medium supplemented with 5 μg/mL trypsin (Biochannel, Nanjing, China) and 37.5 μg/mL pancreatin (Sigma, USA). A mouse monoclonal antibody targeting the PEDV N protein was prepared and preserved in our laboratory. FITC-conjugated AffiniPure goat anti-mouse IgG was purchased from Boster Biological Technology (Wuhan, China).

### Preparation of PEDV-positive swine and rabbit hyperimmune sera

Nine 1-month-old piglets and nine 4- to 6-month-old New Zealand White rabbits were used, and each animal species was randomly divided into three groups (*n* = 3). The JS2008 and AH2012/12 strains were inactivated with β-propiolactone and mixed with Gel-02 adjuvant at a 4:1 volume ratio to prepare inactivated vaccines. Immunized animals received 2 mL of the corresponding inactivated vaccine intramuscularly, and control animals were injected with an equal volume of DMEM. Booster immunizations were given at 14 and 28 days post-vaccination (dpv). Blood samples were collected at 42 dpv for sera preparation and cross-neutralization assays.

### Cross-neutralization assay for PEDV parental and recombinant strains

Neutralization titers of the sera against the virus were assessed according to previously described protocols (47). Briefly, the viruses were adjusted to 200 TCID₅₀/100 μL. Heat-inactivated rabbit hyperimmune sera were serially diluted twofold and incubated with equal volumes of virus at 37°C for 1 h. The mixtures were added to Vero cell monolayers and incubated for 1.5 h. After washing, viral maintenance medium was added, and the cells were cultured for 3–5 days. CPEs were observed under a microscope, and the highest serum dilution that inhibited viral infection by 50% was defined as the neutralizing titer.

On this basis, we further investigated the effects of hyperimmune sera on the stages of viral adsorption and internalization. Purified serum was first diluted into four gradients (100, 50, 25, and 12.5 μg/mL) and incubated with 100 TCID_50_ of virus at 37°C for 1 h. The mixtures were then added to the cells and incubated at 37°C for 1 h. After two washes with phosphate-buffered saline (PBS), virus was added, cells were cultured in maintenance medium for 24 h, and infection was detected by immunofluorescence assay (IFA). The NT_80_ value was defined as the highest serum dilution that reduced viral infectivity by 80% and was used as the working concentration of the neutralizing serum. For the adsorption assay, a mixture of virus (100 TCID_50_) with neutralized sera (1×NT_80_) was preincubated at 37°C for 1 h, then added to the cells and incubated at 4°C for 1 h. After incubation, the cells were washed with PBS before virus quantification. For the internalization assay, 100 TCID_50_ of virus was adsorbed onto the cells at 4°C for 1 h. After washing with PBS, 1×NT_80_ sera were added, and the cells were incubated at 37°C for 1 h. They were then washed with PBS and subsequently quantified. In addition, both the adsorption and internalization experiments were conducted under the respective conditions, followed by IFA to further assess the inhibitory effects.

### Construction of recombinant plasmids

In this study, the infectious clone plasmid pBAC-AH2012/12, which contains the full-length cDNA of the PEDV AH2012/12 strain, was constructed and preserved in our laboratory (47). On the basis of pBAC-AH2012/12, a series of recombinant plasmids were generated via CRISPR/Cas9 technology, in which different segments of the S gene from the JS2008 strain (such as the S, S1, S2, and D0 regions) were inserted into the backbone plasmid. The sequences of primers used in this study are listed in Table S1. The specific amino acid substitutions in each recombinant virus compared with the parental AH2012/12 are summarized in Table S2. Taking pBAC-AH2012/12-893-994aa_JS2008_ as an example, two sgRNAs targeting the 893–993 aa of AH2012/12 were designed and transcribed *in vitro* to produce sgRNAs, which were then used together with Cas9 nuclease to cleave pBAC-AH2012/12. The target replacement fragment was amplified from JS2008 cDNA and subsequently cloned into the linearized pBAC-AH2012/12 backbone via homologous recombination, followed by transformation into 10-beta cells. The successful construction of the recombinant plasmid was confirmed by sequencing.

### Rescue and identification of recombinant viruses

Recombinant plasmids were transfected into Vero cells using Lipofectamine 3000 (Invitrogen) according to the manufacturer’s instructions. After 6–8 h of transfection, the cells were washed with DMEM, and the maintenance medium was replaced with DMEM containing 5 μg/mL trypsin and 37.5 μg/mL pancreatin, or DMEM supplemented with 2% FBS. The cell plates were then incubated at 37°C with 5% CO₂ for 3–5 days until the CPE was observed under a microscope. Successfully rescued recombinant viruses were collected and labeled passage 0 (P0), followed by serial passaging in Vero cells up to passage 5 (P5).

### Indirect IFA

The parental strains JS2008 and AH2012/12, along with the recombinant viruses, were inoculated into monolayer Vero cells at a multiplicity of infection (MOI) of 0.1. After 24 h of infection, the cells were fixed with 4% paraformaldehyde, permeabilized using precooled methanol, and blocked with 5% bovine serum albumin (BSA). After washing the cells twice with PBS, they were incubated with a monoclonal antibody against the PEDV N protein as the primary antibody and FITC-conjugated goat anti-mouse IgG as the secondary antibody. The cell nuclei were stained with 4′,6-diamidino-2-phenylindole (DAPI), followed by two additional washes. The fluorescent signals were visualized and recorded via a fluorescence microscope (Nikon).

### Plaque formation assay

The parental strains JS2008 and AH2012/12, as well as the recombinant viruses, were serially diluted 10-fold six times. For each dilution, 500 μL of the virus suspension was added to a monolayer of Vero cells and incubated at 37°C for 1.5 h. After incubation, the cells were washed twice with DMEM and overlaid with covering medium containing either 1.5% methylcellulose supplemented with 5 μg/mL trypsin and 37.5 μg/mL pancreatin or 1.5% methylcellulose containing 2% FBS. After 3 days of infection, the overlay medium was removed, and the cells were fixed with 4% paraformaldehyde, followed by 0.1% crystal violet staining to visualize and quantify the plaque size and morphology.

### Viral growth kinetics

The parental strains JS2008 and AH2012/12, along with the recombinant viruses, were inoculated onto monolayers of Vero cells at 0.1 MOI for 1.5 h, in the presence or absence of trypsin. The supernatants were collected at 6, 12, 24, 36, 48, and 60 h post-infection (hpi) to measure the viral titers. Growth curves were plotted to evaluate the replication kinetics of different viral strains.

### PRNT for parental and recombinant viruses

Vero cell monolayers in 24-well plates were inoculated with 10-fold serial dilutions of each virus to determine the optimal infection dose (40–60 PFU/well). For the experimental group, 500 μL of twofold serially diluted rabbit hyperimmune sera against JS2008 and AH2012/12 were added. In the control group, 500 μL of DMEM was used as the medium. Subsequently, 500 μL of the optimal virus dilution (40–60 PFU/well) was added and incubated at 37°C for 1 h. Following incubation, the sera-virus mixture was inoculated onto Vero cell monolayers in 24-well plates and incubated at 37°C for 1.5 h. After the unbound viruses were removed by washing with DMEM, an overlay medium was added: trypsin-dependent strains received 1.5% methylcellulose containing 5 μg/mL trypsin and 37.5 μg/mL pancreatin, whereas trypsin-independent strains received 1.5% methylcellulose with 2% FBS. Cells were incubated at 37°C with 5% CO₂ for 3 days. After incubation, the overlay medium was removed, and the cells were fixed with 4% paraformaldehyde and stained with 0.1% crystal violet. Finally, the number of plaques was counted, and the viral inhibition rate was calculated, with 80% plaque reduction defined as the neutralizing titer.

### Pathogenicity assessment of the recombinant viruses

To evaluate the pathogenicity of different recombinant viruses, 35 three-day-old piglets born from sows seronegative for PEDV, TGEV, and PDCoV were randomly divided into seven groups (five piglets per group). Groups 1–6 served as the challenge groups, in which piglets were orally inoculated with 2 mL (5 × 10⁵ TCID₅₀/mL) of the parental or recombinant strains, including rAH2012/12, JS2008, r-S, r-S1, r-S2, and r-894-993aa. Group 7 served as the negative control and received 2 mL of DMEM. Following the viral challenge, all piglets were clinically monitored, including assessments of diarrhea, vomiting, and mental state. Diarrhea severity was scored based on fecal consistency, with scores of 0 (solid), 1 (paste-like), 2 (semiliquid), and 3 (watery). To assess viral shedding, rectal swabs were collected daily from the day of the challenge. Mortality was recorded throughout the study, and three and two piglets from each group were euthanized and necropsied at 3 and 5 dpc, respectively. During necropsy, intestinal tissues were photographed and collected for quantification of the PEDV viral load and histopathological examination to assess the degree of intestinal lesions.

### Evaluation of the immunogenicity and protective efficacy of rAH2012/12 and r-894-993aa

To investigate the immunogenicity and protective efficacy of the rAH2012/12 and r-894-993aa inactivated vaccines, 20 seven-day-old PEDV, TGEV, and PDCoV seronegative piglets were randomly assigned to four groups: rAH2012/12 immunized, r-894-993aa immunized, challenge control, and negative control. Piglets in the immunized groups were injected with 2 mL of 10^6^ TCID_50_/mL inactivated vaccine, whereas those in the challenge control group received 2 mL of DMEM with adjuvant. Primary and booster immunizations were administered at 7 and 14 days of age, respectively. Serum samples were collected at 0 and 24 days post-vaccination (dpv) to determine antibody titers and cross-neutralization activity. At 24 dpv, piglets in the vaccinated and challenge control groups were orally challenged with 5 × 10⁵ TCID₅₀/30 mL of the rAH2012/12 strain. In accordance with the previous methods, clinical signs were monitored for 15 consecutive days following viral challenge (47). Necropsy and tissue sampling were performed at the time of piglet death or trial completion to assess intestinal lesions and quantify viral loads via RT-qPCR targeting the conserved region of the PEDV N gene using specific primers and probes (Table S1).

### Enzyme-linked immunosorbent assay

An indirect ELISA was performed to detect serum antibody levels against the virus or the PEDV-S1 protein. The virus (200 TCID₅₀) or PEDV- S1 protein (0.25 μg/mL) was coated onto 96-well plates (100 μL/well) and incubated overnight at 4°C. After blocking with 5% skim milk at 37°C for 3 h, the plates were washed and incubated with 1:100-diluted sera at 37°C for 30 min. Following washing, an HRP-conjugated secondary antibody (1:10,000) was added, and the mixture was incubated at 37°C in the dark for 30 min. Finally, the TMB solution was added to each well, and the plates were incubated at 37°C in the dark for 10 min. The reaction was terminated by adding the stop solution. The absorbance was measured at 450 nm via an ELISA reader.

### Construction of cell-free and cell-to-cell viral transmission models and evaluation of resistance to immune sera

To investigate cell-free and cell-to-cell viral transmission, Vero cells were infected with JS2008, AH2012/12, or r-894-993aa at an MOI of 0.1. At 8 hpi, the infected cells were harvested via enzymatic digestion and collected as donor cells. These donor cells were co-seeded with uninfected target cells at a 1:3 ratio in 24-well plates, forming a mixed infection model that involved both cell-free and cell-to-cell transmission. To model cell-free infection, equivalent numbers of donor cells were inoculated into 0.4 μm pore-size Transwell inserts (SAINING), which separate donor and target cells while allowing only mature viral particles to pass through. After 6, 12, 18, and 24 h of coculture, all cells of the mixed infection model, as well as the cells above and below the Transwell inserts, were collected. Viral RNA copy numbers were quantified via RT-quantitative PCR (RT-qPCR). The amount of cell-to-cell transmission was estimated by subtracting the viral copy numbers in the cell-free model and the donor cells in the upper Transwell inserts from the total viral load in the mixed infection model.

To assess the neutralizing efficacy of anti-JS2008 immune sera in different transmission models, infection models were established as described above. Purified immune sera at a concentration of 1 × NT₅₀, along with an equal concentration of negative control sera, were added, and the cells were cultured at 37°C for 24 h.

### Structural modeling and analysis

The amino acid sequences of the S proteins of the JS2008 and AH2012/12 strains were submitted to SWISS-MODEL for homology modeling to generate three-dimensional protein structures. The models with the highest global model quality estimation (GMQE) and QMEAN scores were selected for subsequent analyses. To evaluate the impact of key amino acid mutations on the physicochemical properties of the S protein surface, isoelectric points (pIs) and side chain polarity were analyzed. The protein electrostatic potential and hydrophilicity/hydrophobicity were visualized via PyMOL. Additionally, structural alignment was performed to compare the overall conformations of the S proteins from the JS2008 and AH2012/12 strains. Furthermore, the interactions between key amino acid residues and surrounding molecules within a 5 Å radius were computed to assess potential effects on protein stability and function.

### Statistical analysis

Statistical analyses were performed on sera cross-neutralization titers, plaque sizes, PRNT results, tissue viral loads, and sera antibody titers. The results are expressed as the mean ± standard deviation (SD). Data visualization and statistical analyses were performed with GraphPad Prism 8.0. Data normality was assessed using the Shapiro-Wilk normality test, and homogeneity of variances was examined by the Brown-Forsythe test. Comparisons between two groups were performed using a two-tailed Student’s *t* test or nonparametric tests, as appropriate. Comparisons among multiple groups were conducted using one-way analysis of variance (ANOVA) or Welch’s ANOVA, followed by multiple comparisons between groups. Non-normally distributed data were analyzed using nonparametric methods. Significance was denoted as follows: *P* < 0.05 (*), *P* < 0.01 (**), *P* < 0.001 (***), *P* < 0.001 (****), and *P* ≥ 0.05 indicates no significance (NS). For multiple group comparisons, different letters (such as a, b, and c) indicate statistically significant differences (*P* < 0.05) between groups, whereas the same letter indicates no statistically significant difference (*P* > 0.05) (57). All assays were conducted in triplicate or more.

## Data Availability

All data used for statistical analyses and to generate graphs have been uploaded in figshare with URL https://figshare.com/s/4972011039d7df13325a.

## SUPPLEMENTAL MATERIAL

Supplemental Material mbio.00026-26-s0001.docx Tables S1 and S2 and Figures S1-S6.

ASM does not own the copyrights to Supplemental Material that may be linked to, or accessed through, an article. The authors have granted ASM a non-exclusive, world-wide license to publish the Supplemental Material files. Please contact the corresponding author directly for reuse.

## References

[1] Jung K, Saif LJ, Wang Q. 2020. Porcine epidemic diarrhea virus (PEDV): an update on etiology, transmission, pathogenesis, and prevention and control. Virus Res 286:198045. doi:10.1016/j.virusres.2020.198045 32502552 PMC7266596

[2] Sueyoshi M, Tsuda T, Yamazaki K, Yoshida K, Nakazawa M, Sato K, Minami T, Iwashita K, Watanabe M, Suzuki Y. 1995. An immunohistochemical investigation of porcine epidemic diarrhoea. J Comp Pathol 113:59–67. doi:10.1016/s0021-9975(05)80069-6 7490338 PMC7130422

[3] Pensaert MB, de Bouck P. 1978. A new coronavirus-like particle associated with diarrhea in swine. Arch Virol 58:243–247. doi:10.1007/BF01317606 83132 PMC7086830

[4] Debouck P, Pensaert M. 1980. Experimental infection of pigs with a new porcine enteric coronavirus, CV 777. Am J Vet Res 41:219–223. doi:10.2460/ajvr.1980.41.02.219 6245603

[5] Li W, Li H, Liu Y, Pan Y, Deng F, Song Y, Tang X, He Q. 2012. New variants of porcine epidemic diarrhea virus, China, 2011. Emerg Infect Dis 18:1350–1353. doi:10.3201/eid1808.120002 22840964 PMC3414035

[6] Sun D, Wang X, Wei S, Chen J, Feng L. 2016. Epidemiology and vaccine of porcine epidemic diarrhea virus in China: a mini-review. J Vet Med Sci 78:355–363. doi:10.1292/jvms.15-0446 26537549 PMC4829501

[7] Ojkic D, Hazlett M, Fairles J, Marom A, Slavic D, Maxie G, Alexandersen S, Pasick J, Alsop J, Burlatschenko S. 2015. The first case of porcine epidemic diarrhea in Canada. Can Vet J 56:149–152. 25694663 PMC4298265

[8] Lara-Romero R, Gómez-Núñez L, Cerriteño-Sánchez JL, Márquez-Valdelamar L, Mendoza-Elvira S, Ramírez-Mendoza H, Rivera-Benítez JF. 2018. Molecular characterization of the spike gene of the porcine epidemic diarrhea virus in Mexico, 2013–2016. Virus Genes 54:215–224. doi:10.1007/s11262-017-1528-x 29243063 PMC7088687

[9] Cima G. 2014. PED virus reinfecting U.S. herds. Virus estimated to have killed 7 million-plus pigs. J Am Vet Med Assoc 245:166–167. 25115019

[10] Sun RQ, Cai RJ, Chen YQ, Liang PS, Chen DK, Song CX. 2012. Outbreak of porcine epidemic diarrhea in suckling piglets, China. Emerg Infect Dis 18:161–163. doi:10.3201/eid1801.111259 22261231 PMC3381683

[11] Lin F, Zhang H, Li L, Yang Y, Zou X, Chen J, Tang X. 2022. PEDV: insights and advances into types, function, structure, and receptor recognition. Viruses 14:1744. doi:10.3390/v14081744 36016366 PMC9416423

[12] Li X, Wu Y, Yan Z, Li G, Luo J, Huang S, Guo X. 2024. A comprehensive view on the protein functions of porcine epidemic diarrhea virus. Genes (Basel) 15:165. doi:10.3390/genes15020165 38397155 PMC10887554

[13] Guo J, Fang L, Ye X, Chen J, Xu S, Zhu X, Miao Y, Wang D, Xiao S. 2019. Evolutionary and genotypic analyses of global porcine epidemic diarrhea virus strains. Transbound Emerg Dis 66:111–118. doi:10.1111/tbed.12991 30102851 PMC7168555

[14] Zhang Y, Chen Y, Zhou J, Wang X, Ma L, Li J, Yang L, Yuan H, Pang D, Ouyang H. 2022. Porcine epidemic diarrhea virus: an updated overview of virus epidemiology, virulence variation patterns and virus-host interactions. Viruses 14:2434. doi:10.3390/v14112434 36366532 PMC9695474

[15] Peng Q, Fu P, Zhou Y, Lang Y, Zhao S, Wen Y, Wang Y, Wu R, Zhao Q, Du S, Cao S, Huang X, Yan Q. 2024. Phylogenetic analysis of porcine epidemic diarrhea virus (PEDV) during 2020–2022 and isolation of a variant recombinant PEDV strain. Int J Mol Sci 25:10878. doi:10.3390/ijms252010878 39456662 PMC11507624

[16] Lin CM, Saif LJ, Marthaler D, Wang Q. 2016. Evolution, antigenicity and pathogenicity of global porcine epidemic diarrhea virus strains. Virus Res 226:20–39. doi:10.1016/j.virusres.2016.05.023 27288724 PMC7111424

[17] Zhang Y, Chen Y, Yuan W, Peng Q, Zhang F, Ye Y, Huang D, Ding Z, Lin L, He H, Wu Q, Song D, Tang Y. 2020. Evaluation of cross-protection between G1a- and G2a-genotype porcine epidemic diarrhea viruses in suckling piglets. Animals (Basel) 10:1674. doi:10.3390/ani10091674 32957461 PMC7552732

[18] Bosch BJ, van der Zee R, de Haan CAM, Rottier PJM. 2003. The coronavirus spike protein is a class I virus fusion protein: structural and functional characterization of the fusion core complex. J Virol 77:8801–8811. doi:10.1128/jvi.77.16.8801-8811.2003 12885899 PMC167208

[19] Lee DK, Park CK, Kim SH, Lee C. 2010. Heterogeneity in spike protein genes of porcine epidemic diarrhea viruses isolated in Korea. Virus Res 149:175–182. doi:10.1016/j.virusres.2010.01.015 20132850 PMC7114470

[20] Li W, van Kuppeveld FJM, He Q, Rottier PJM, Bosch B-J. 2016. Cellular entry of the porcine epidemic diarrhea virus. Virus Res 226:117–127. doi:10.1016/j.virusres.2016.05.031 27317167 PMC7114534

[21] Mao L, Cai X, Li J, Li X, Li S, Li W, Lu H, Dong Y, Zhai J, Xu X, Li B. 2025. Discovery of a novel Betacoronavirus 1, cpCoV, in goats in China: the new risk of cross-species transmission. PLoS Pathog 21:e1012974. doi:10.1371/journal.ppat.1012974 40100842 PMC11918373

[22] Oh J, Lee KW, Choi HW, Lee C. 2014. Immunogenicity and protective efficacy of recombinant S1 domain of the porcine epidemic diarrhea virus spike protein. Arch Virol 159:2977–2987. doi:10.1007/s00705-014-2163-7 25008896 PMC7086977

[23] Okda FA, Lawson S, Singrey A, Nelson J, Hain KS, Joshi LR, Christopher-Hennings J, Nelson EA, Diel DG. 2017. The S2 glycoprotein subunit of porcine epidemic diarrhea virus contains immunodominant neutralizing epitopes. Virology (Auckland) 509:185–194. doi:10.1016/j.virol.2017.06.013 PMC7111671 28647506

[24] Harvey WT, Carabelli AM, Jackson B, Gupta RK, Thomson EC, Harrison EM, Ludden C, Reeve R, Rambaut A, COVID-19 Genomics UK (COG-UK) Consortium, Peacock SJ, Robertson DL. 2021. SARS-CoV-2 variants, spike mutations and immune escape. Nat Rev Microbiol 19:409–424. doi:10.1038/s41579-021-00573-0 34075212 PMC8167834

[25] Thomson EC, Rosen LE, Shepherd JG, Spreafico R, da Silva Filipe A, Wojcechowskyj JA, Davis C, Piccoli L, Pascall DJ, Dillen J, et al.. 2021. Circulating SARS-CoV-2 spike N439K variants maintain fitness while evading antibody-mediated immunity. Cell 184:1171–1187. doi:10.1016/j.cell.2021.01.037 33621484 PMC7843029

[26] Zhang H, Han F, Yan X, Liu L, Shu X, Hu H. 2021. Prevalence and phylogenetic analysis of spike gene of porcine epidemic diarrhea virus in Henan province, China in 2015–2019. Infect Genet Evol 88:104709. doi:10.1016/j.meegid.2021.104709 33412288

[27] Chen F, Ku X, Li Z, Memon AM, Ye S, Zhu Y, Zhou C, Yao L, Meng X, He Q. 2016. Genetic characteristics of porcine epidemic diarrhea virus in Chinese mainland, revealing genetic markers of classical and variant virulent parental/attenuated strains. Gene 588:95–102. doi:10.1016/j.gene.2016.05.011 27178127

[28] Wang X, Chen J, Shi D, Shi H, Zhang X, Yuan J, Jiang S, Feng L. 2016. Immunogenicity and antigenic relationships among spike proteins of porcine epidemic diarrhea virus subtypes G1 and G2. Arch Virol 161:537–547. doi:10.1007/s00705-015-2694-6 26611909 PMC7087089

[29] Kumar N, Bajiya N, Patiyal S, Raghava GPS. 2023. Multi-perspectives and challenges in identifying B-cell epitopes. Protein Sci 32:e4785. doi:10.1002/pro.4785 37733481 PMC10578127

[30] Zobayer N, Hossain AA, Rahman MA. 2019. A combined view of B-cell epitope features in antigens. Bioinformation 15:530–534. doi:10.6026/97320630015530 31485139 PMC6704334

[31] Chen Q, Li G, Stasko J, Thomas JT, Stensland WR, Pillatzki AE, Gauger PC, Schwartz KJ, Madson D, Yoon KJ, Stevenson GW, Burrough ER, Harmon KM, Main RG, Zhang J. 2014. Isolation and characterization of porcine epidemic diarrhea viruses associated with the 2013 disease outbreak among swine in the United States. J Clin Microbiol 52:234–243. doi:10.1128/JCM.02820-13 24197882 PMC3911415

[32] Lee C. 2015. Porcine epidemic diarrhea virus: an emerging and re-emerging epizootic swine virus. Virol J 12:193. doi:10.1186/s12985-015-0421-2 26689811 PMC4687282

[33] Li C, Li W, Lucio de Esesarte E, Guo H, van den Elzen P, Aarts E, van den Born E, Rottier PJM, Bosch B-J. 2017. Cell attachment domains of the porcine epidemic diarrhea virus spike protein are key targets of neutralizing antibodies. J Virol 91:e00273-17. doi:10.1128/JVI.00273-17 28381581 PMC5446644

[34] Kirchdoerfer RN, Bhandari M, Martini O, Sewall LM, Bangaru S, Yoon KJ, Ward AB. 2021. Structure and immune recognition of the porcine epidemic diarrhea virus spike protein. Structure 29:385–392. doi:10.1016/j.str.2020.12.003 33378641 PMC7962898

[35] Godet M, Grosclaude J, Delmas B, Laude H. 1994. Major receptor-binding and neutralization determinants are located within the same domain of the transmissible gastroenteritis virus (coronavirus) spike protein. J Virol 68:8008–8016. doi:10.1128/JVI.68.12.8008-8016.1994 7525985 PMC237264

[36] Cruz DJM, Kim C-J, Shin H-J. 2008. The GPRLQPY motif located at the carboxy-terminal of the spike protein induces antibodies that neutralize Porcine epidemic diarrhea virus. Virus Res 132:192–196. doi:10.1016/j.virusres.2007.10.015 18067984 PMC7114191

[37] Tan Y, Sun L, Wang G, Shi Y, Dong W, Fu Y, Fu Z, Chen H, Peng G. 2021. The trypsin-enhanced infection of porcine epidemic diarrhea virus is determined by the S2 subunit of the spike glycoprotein. J Virol 95:e02453-20. doi:10.1128/JVI.02453-20 33692210 PMC8139691

[38] Li M, Zhang Y, Fang Y, Xiao S, Fang P, Fang L. 2023. Construction and immunogenicity of a trypsin-independent porcine epidemic diarrhea virus variant. Front Immunol 14:1165606. doi:10.3389/fimmu.2023.1165606 37033982 PMC10080105

[39] Zeng C, Evans JP, King T, Zheng YM, Oltz EM, Whelan SPJ, Saif LJ, Peeples ME, Liu SL. 2022. SARS-CoV-2 spreads through cell-to-cell transmission. Proc Natl Acad Sci USA 119:e2111400119. doi:10.1073/pnas.2111400119 34937699 PMC8740724

[40] Xia S, Xiao W, Zhu X, Liao S, Guo J, Zhou J, Xiao S, Fang P, Fang L. 2023. Porcine deltacoronavirus resists antibody neutralization through cell-to-cell transmission. Emerg Microbes Infect 12:2207688. doi:10.1080/22221751.2023.2207688 37125733 PMC10193906

[41] Tortorici MA, Choi A, Gibson CA, Lee J, Brown JT, Stewart C, Joshi A, Harari S, Willoughby I, Treichel C, Leaf EM, Bloom JD, King NP, Tait-Burkard C, Whittaker GR, Veesler D. 2025. Loss of FCoV-23 spike domain 0 enhances fusogenicity and entry kinetics. Nature 645:235–243. doi:10.1038/s41586-025-09155-z 40634609 PMC12408340

[42] Jang G, Min KC, Lee IH, Won H, Yoon IJ, Kang SC, Lee C. 2023. Deletion of pentad residues in the N-terminal domain of spike protein attenuates porcine epidemic diarrhea virus in piglets. Vet Microbiol 280:109727. doi:10.1016/j.vetmic.2023.109727 36958068

[43] Ma Z, Li Z, Li Y, Zhao X, Zheng C, Li Y, Guo X, Xu L, Zheng Z, Liu G, Zheng H, Xiao S. 2025. Changes in the motifs in the D0 and SD2 domains of the S protein drive the evolution of virulence in enteric coronavirus porcine epidemic diarrhea virus. J Virol 99:e02092-24. doi:10.1128/jvi.02092-24 40035514 PMC11998522

[44] Zhang D, Xie Y, Liao Q, Jiao Z, Liang R, Zhang J, Zhang Y, Tan Y, Wang H, Zhang W, Xiao S, Peng G, Shi Y. 2024. Development of a safe and broad-spectrum attenuated PEDV vaccine candidate by S2 subunit replacement. J Virol 98:e00429-24. doi:10.1128/jvi.00429-24 39404450 PMC11575183

[45] Hou Y, Meulia T, Gao X, Saif LJ, Wang Q. 2019. Deletion of both the tyrosine-based endocytosis signal and the endoplasmic reticulum retrieval signal in the cytoplasmic tail of spike protein attenuates porcine epidemic diarrhea virus in pigs. J Virol 93:e01758-18. doi:10.1128/JVI.01758-18 30404797 PMC6321913

[46] Song S, Park GN, Shin J, Kim KS, An BH, Choe S, Kim SY, Hyun BH, An DJ. 2023. Rescue of a live-attenuated porcine epidemic diarrhea virus HSGP strain using a virulent strain and a partially attenuated strain. Viruses 15:1601. doi:10.3390/v15071601 37515287 PMC10383568

[47] Peng Q, Fan B, Song X, He W, Wang C, Zhao Y, Guo W, Zhang X, Liu S, Gao J, Li K, Zhang B, Zhou J, Li Y, Guo R, Li B. 2023. Genetic signatures associated with the virulence of porcine epidemic diarrhea virus AH2012/12. J Virol 97:e01063-23. doi:10.1128/jvi.01063-23 37732788 PMC10617547

[48] Gao X, Yu B, Yu J, Mao X, Huang Z, Luo Y, Luo J, Zheng P, Yan H, He J, Chen D. 2022. Developmental profiling of dietary carbohydrate digestion in piglets. Front Microbiol 13:896660. doi:10.3389/fmicb.2022.896660 35572714 PMC9100932

[49] Wicht O, Li W, Willems L, Meuleman TJ, Wubbolts RW, van Kuppeveld FJM, Rottier PJM, Bosch BJ. 2014. Proteolytic activation of the porcine epidemic diarrhea coronavirus spike fusion protein by trypsin in cell culture. J Virol 88:7952–7961. doi:10.1128/JVI.00297-14 24807723 PMC4097775

[50] Ye C, Chiem K, Park J-G, Oladunni F, Platt RN II, Anderson T, Almazan F, de la Torre JC, Martinez-Sobrido L. 2020. Rescue of SARS-CoV-2 from a single bacterial artificial chromosome. mBio 11:e02168-20. doi:10.1128/mBio.02168-20 32978313 PMC7520601

[51] Li Z, Ma Z, Dong L, Yang T, Li Y, Jiao D, Han W, Zheng H, Xiao S. 2022. Molecular mechanism of porcine epidemic diarrhea virus cell tropism. mBio 13:e03739-21. doi:10.1128/mbio.03739-21 35285698 PMC9040822

[52] Fan X, Cao D, Kong L, Zhang X. 2020. Cryo-EM analysis of the post-fusion structure of the SARS-CoV spike glycoprotein. Nat Commun 11:3618. doi:10.1038/s41467-020-17371-6 32681106 PMC7367865

[53] Petit CM, Melancon JM, Chouljenko VN, Colgrove R, Farzan M, Knipe DM, Kousoulas KG. 2005. Genetic analysis of the SARS-coronavirus spike glycoprotein functional domains involved in cell-surface expression and cell-to-cell fusion. Virology (Auckland) 341:215–230. doi:10.1016/j.virol.2005.06.046 PMC7111838 16099010

[54] Yang K, Wang C, White KI, Pfuetzner RA, Esquivies L, Brunger AT. 2022. Structural conservation among variants of the SARS-CoV-2 spike postfusion bundle. Proc Natl Acad Sci USA 119:e2119467119. doi:10.1073/pnas.2119467119 35363556 PMC9169775

[55] Mannar D, Saville JW, Sun Z, Zhu X, Marti MM, Srivastava SS, Berezuk AM, Zhou S, Tuttle KS, Sobolewski MD, Kim A, Treat BR, Da Silva Castanha PM, Jacobs JL, Barratt-Boyes SM, Mellors JW, Dimitrov DS, Li W, Subramaniam S. 2022. SARS-CoV-2 variants of concern: spike protein mutational analysis and epitope for broad neutralization. Nat Commun 13:4696. doi:10.1038/s41467-022-32262-8 35982054 PMC9388680

[56] Lyukmanova EN, Pichkur EB, Nolde DE, Kocharovskaya MV, Manuvera VA, Shirokov DA, Kharlampieva DD, Grafskaia EN, Svetlova JI, Lazarev VN, Varizhuk AM, Kirpichnikov MP, Shenkarev ZO. 2024. Structure and dynamics of the interaction of Delta and Omicron BA.1 SARS-CoV-2 variants with REGN10987 Fab reveal mechanism of antibody action. Commun Biol 7:1698. doi:10.1038/s42003-024-07422-9 39719448 PMC11668877

[57] Piepho H-P. 2004. An algorithm for a letter-based representation of all-pairwise comparisons. J Comput Graph Stat 13:456–466. doi:10.1198/1061860043515

