# Supplementary material for: Evasion of humoral immune responses by a key mutational region of the S2 subunit in PEDV variants
Source: mBio. 2026 Mar 23;17(4):e00026-26. doi: 10.1128/mbio.00026-26 (PMC13059768; doi:10.1128/mbio.00026-26)
Supplement: Supplemental Material — Tables S1 and S2 and Figures S1-S6. [file mbio.00026-26-s0001.docx]

Table S1. Primers used in this paper

| Primer | Sequence (5’-3’) |
| --- | --- |
| sgRNA-S-F | TTCTAATACGACTCACTATAGGTTGTGGTTTTTCTAATCATTGTTTTAGAGCTAGA |
| sgRNA-S-R | TTCTAATACGACTCACTATAGGCCACGTGCAGTGATGTTTCTGTTTTAGAGCTAGA |
| S-F | CATTTGTGGTTTTTCTAATC |
| S-R | TGTATTGAAAAAGTCCAAGA |
| sgRNA-S1-F | TTCTAATACGACTCACTATAGGTTGTGGTTTTTCTAATCATTGTTTTAGAGCTAGA |
| sgRNA-S1-R | TTCTAATACGACTCACTATAGGCCCTGTACAGCTAATTGCATGTTTTAGAGCTAGA |
| S1-F | CATTTGTGGTTTTTCTAATCATTTGG |
| S1-R | TGTAAATATTCTGTCCTAATACTCATACTAAAGTTGGTGG |
| sgRNA-S2-F | TTCTAATACGACTCACTATAGGCCAACTTTAGTATGAGTATTGTTTTAGAGCTAGA |
| sgRNA-S2-R | TTCTAATACGACTCACTATAGGCCACGTGCAGTGATGTTTCTGTTTTAGAGCTAGA |
| S2-F | CCACCAACTTTAGTATGAGT |
| S2-R | TGTATTGAAAAAGTCCAAGA |
| sgRNA-D0-F | TTCTAATACGACTCACTATAGGTTGTGGTTTTTCTAATCATTGTTTTAGAGCTAGA |
| sgRNA-D0-R | TTCTAATACGACTCACTATAGGCCCTGTACAGCTAATTGCATGTTTTAGAGCTAGA |
| D0-F | CATTTGTGGTTTTTCTAATCATTTGG |
| D0-R | CATTGGCAGCATAACCACTG |
| sgRNA-D1-F | TTCTAATACGACTCACTATAGGTGATGACACCATTAATATGTTTTAGAGCTAGA |
| sgRNA-D1-R | TTCTAATACGACTCACTATAGGGCTCAGTAGCAAATACATGTTTTAGAGCTAGA |
| D1-upF | AAGAAGGCTTCTTTCCCTATA |
| D1-upR | CTAAAGTTAATAGTAGACTGGCACCTGGTGACATCTTGTG |
| D1-middleF | CAGTCTACTATTAACTTTAGG |
| D1-middleR | CTGACCAGAATCGATGTAACT |
| D1-downF | AGTTACATCGATTCTGGTCAGGGCTTTGAGATTGGCATTTC |
| D1-downR | CATTGGGCTCAGTAGCAAATA |
| sgRNA-D2-F | TTCTAATACGACTCACTATAGGTGTTAGGTTTGTTGAAGAAGTTTTAGAGCTAGA |
| sgRNA-D2-R | TTCTAATACGACTCACTATAGGTTTCCAACCAACCATTGTGTTTTAGAGCTAGA |
| D2-upF | GTTGTGTTAGGTTTGTTGAA |
| D2-upR | CAAATGCGCAGTCGTGCAATAGCATTAGTGTTACCGTTAG |
| D2-middleF | ATTGCACGACTGCGCATTTG |
| D2-middleR | AATATTTTTTCCATCCTGCA |
| D2-downF | TGCAGGATGGAAAAAATATTGTTGTCGGCATAACATGGGA |
| D2-downR | CAAAAGACAATTGACCAACA |
| sgRNA-D3-F | TTCTAATACGACTCACTATAGGCGCCTGCAGTTGTTGTACGTTTTAGAGCTAGA |
| sgRNA-D3-R | TTCTAATACGACTCACTATAGGTGATGACAATTTCCCTGAGTTTTAGAGCTAGA |
| D3-upF | TCAGGCGCCTGCAGTTGTTG |
| D3-upR | AAATGATAGATCTTGTCAGCAAAGACAGTGACACGATCAT |
| D3-middleF | GCTGACAAGATCTATCATTT |
| D3-middleR | ACTGCAATTAGCTGTACAAG |
| D3-downF | CTTGTACAGCTAATTGCAGTGGTTATGCTGCCAATGTATT |
| D3-downR | CTTGGTGATGACAATTTCCC |
| sgRNA-D4-F | TTCTAATACGACTCACTATAGGCCCTGTACAGCTAATTGCATGTTTTAGAGCTAGA |
| sgRNA-D4-R | TTCTAATACGACTCACTATAGGCCAACTTTAGTATGAGTATTGTTTTAGAGCTAGA |
| D4-F | CAACCCTGTACAGCTAATTGCATTGGTTACGCTGCCAATGTAT |
| D4-R | TGTAAATATTCTGTCCTAATACTCATACTAAAGTTGGTGG |
| sgRNA-NTD1-F | TTCTAATACGACTCACTATAGGAGCCATATTAGAGGTGGTCAGTTTTAGAGCTAGA |
| sgRNA-NTD1-R | TTCTAATACGACTCACTATAGGAACAAAATTAGTCGATGCTAGTTTTAGAGCTAGA |
| NTD1-upF | CTTAGCCATATTAGAGGTGGTCATGG |
| NTD1-upR | TGCAATTAGCTGTACAGGGTTGAT |
| NTD1-middleF | ACCCTGTACAGCTAATTGCAGTGGTTACG |
| NTD1-middleR | GCTCCATTACAAACACCATCCATCGTT |
| NTD1-downF | CGATGGTGTTTGTAATGGAGCT |
| NTD1-downR | ATCAACAAAATTAGTCGATGCTATGGTC |
| sgRNA-NTD2-F | TTCTAATACGACTCACTATAGGCGACAAAGTGTTACAACAGGTTTTAGAGCTAGA |
| sgRNA-NTD2-R | TTCTAATACGACTCACTATAGGAAAGAATGATCATTAAATGAGTTTTAGAGCTAGA |
| NTD2-upF | GTTGCGACAAAGTGTTACAACAGT |
| NTD2-upR | ATTTGTTCCTAAAGCAGTATGAAGTACAA |
| NTD2-middleF | ATACTGCTTTAGGAACAAATCTTTCTTTTG |
| NTD2-middleR | AGAAAACAATAATAGGGTACTTCAGTAACACCCAA |
| NTD2-downF | GTACCCTATTATTGTTTTCTTAAAGTGGA |
| NTD2-downR | ACAAAAGAATGATCATTAAATGATGGCAAAG |
| sgRNA-CTD-F | TTCTAATACGACTCACTATAGGAAATTGTCATCACCAAGTAGTTTTAGAGCTAGA |
| sgRNA-CTD-R | TTCTAATACGACTCACTATAGGATGTTACTATACACCAACACGTTTTAGAGCTAGA |
| CTD-upF | AGGGAAATTGTCATCACCAAGTATGG |
| CTD-upR | AATGGTAAATTGTCTAGTGTCAACACAG |
| CTD-middleF | ACACTAGACAATTTACCATTACACTGTTT |
| CTD-middleR | AAAAGATCTATGGTACAGGCACCAGCC |
| CTD-downF | GCCTGTACCATAGATCTTTTTGGT |
| CTD-downR | CCTATGTTACTATACACCAACACAGGCT |
| sgRNA-SD2-F | TTCTAATACGACTCACTATAGGCATCTGACACTACTATCAAGTTTTAGAGCTAGA |
| sgRNA-SD2-R | TTCTAATACGACTCACTATAGGATCACCATTAAACGAACTGAGTTTTAGAGCTAGA |
| SD2-upF | ATTGCATCTGACACTACTATCAATGG |
| SD2-upR | AGCTAACAACTGTCCAGAAT |
| SD2-middleF | ATTCTGGACAGTTGTTAGCTTTTAAGAATG |
| SD2-middleR | AAGAAACCAGGCAACTCCCTAGT |
| SD2-downF | AGGGAGTTGCCTGGTTTCTT |
| SD2-downR | TCCATCACCATTAAACGAACTGATGG |
| sgRNA-763-993aa-F | TTCTAATACGACTCACTATAGGTTTGTAAATCTGGCAGTATGTTTTAGAGCTAGA |
| sgRNA-763-993aa-R | TTCTAATACGACTCACTATAGGTACAGACGGATGTTCTACAGGTTTTAGAGCTAGA |
| 763-993aa-F | GGTGTTTGTAAATCTGGCAGTATTGG |
| 763-993aa-R | AGCAATTGCTGGTTCCGCTG |
| sgRNA-994-1302aa-F | TTCTAATACGACTCACTATAGGTACAGACGGATGTTCTACAGGTTTTAGAGCTAGA |
| sgRNA-994-1302aa-R | TTCTAATACGACTCACTATAGGACACACTAGTTGACCTTGAGGTTTTAGAGCTAGA |
| 994-1302aa-F | CTCTACAGACGGATGTTCTACAGCG |
| 994-1302aa-R | TCAACTCGGTTGAGCCACTC |
| sgRNA-763-893aa-F | TTCTAATACGACTCACTATAGGTTTGTAAATCTGGCAGTATGTTTTAGAGCTAGA |
| sgRNA-763-893aa-R | TTCTAATACGACTCACTATAGGACCTGCTTTTTAATAAAGGTTTTAGAGCTAGA |
| 763-893aa-F | GGTGTCTGTAAATCTGGCAGTATTGGC |
| 763-893aa-R | AAGGCCATTAGTAACCACTTTATTAAAAAGC |
| sgRNA-894-993aa-F | TTCTAATACGACTCACTATAGGTGGCAGGGTGGTACAAAAAGTTTTAGAGCTAGA |
| sgRNA-894-993aa-R | TTCTAATACGACTCACTATAGGTACAGACGGATGTTCTACAGGTTTTAGAGCTAGA |
| 894-993aa-F | CAAGTGGCAGGGTGGTACAA |
| 894-993aa-R | AGCAATTGCTGGTTCCGCTG |
| sgRNA-894-993aa-single-F | TTCTAATACGACTCACTATAGGTGTGCTACATATGTTTGTAAGTTTTAGAGCTAGA |
| sgRNA-894-993aa-single-R | TTCTAATACGACTCACTATAGGAACCCTTACTAAGTATACTGGTTTTAGAGCTAGA |
| single-mut-upF | GATTGTGCTACATATGTTTGTAATGGTAACTC |
| R894G-upR | TTTTTGTACCACCCTGCCACT |
| R894G-downF | GTGGCAGGGTGGTACAAAAAGGGTCTTTCATTGAAGACCT |
| single-mut-upR | CCTGCTAGCCTGAACCTCAGTA |
| F966L-upR | ACCTCCTAGCACCATACCACC |
| F966L-downF | GGTATGGTGCTAGGAGGTCTTACTGCAGCAGCGGCA |
| A968T-upR | AGTAAAACCTCCTAGCACCATACCAC |
| A968T-downF | GTGCTAGGAGGTTTTACTACTGCAGCGGCATTGCCT |
| Y976H-upR | GCTAAAAGGCAATGCCGCTG |
| Y976H-downF | GCGGCATTGCCTTTTAGCCATGCTGTTCAAGCGAGA |
| sense primer | GTCTGAAAAGCCAATCATTC |
| antisense primer | TTGCCTCTGTTGTTACTC |
| probe | CTGTTGTTGCCATTGCCACGA |

Table S2. Mutation sites of recombinant viruses compared with the parental AH2012/12

| Recombinant virus | Mutation sites compared to AH2012/12 S protein |
| --- | --- |
| rAH2012/12-S_JS2008_ | 1-1385 aa |
| rAH2012/12-S1_JS2008_ | 1-782 aa |
| rAH2012/12-S2_JS2008_ | 783-1385 aa |
| rAH2012/12-D0_JS2008_ | 27-235 aa |
| rAH2012/12-D1_JS2008_ | 27-89 aa |
| rAH2012/12-D2_JS2008_ | 120-162 aa |
| rAH2012/12-D3_JS2008_ | 177-235 aa |
| rAH2012/12-D4_JS2008_ | 236-782 aa |
| rAH2012/12-NTD1_JS2008_ | 236-303 aa |
| rAH2012/12-NTD2_JS2008_ | 345-368 aa |
| rAH2012/12-CTD_JS2008_ | 552-597 aa |
| rAH2012/12-SD2_JS2008_ | 722-728 aa |
| rAH2012/12-S2-763-893_JS2008_ | 763-893 aa |
| rAH2012/12-S2-894-993_JS2008_ | 894-993 aa |
| rAH2012/12-S2-994-1302_JS2008_ | 994-1302 aa |
| rAH2012/12-R894G | 894 |
| rAH2012/12-F966L | 966 |
| rAH2012/12-A968T | 968 |
| rAH2012/12-Y976H | 976 |


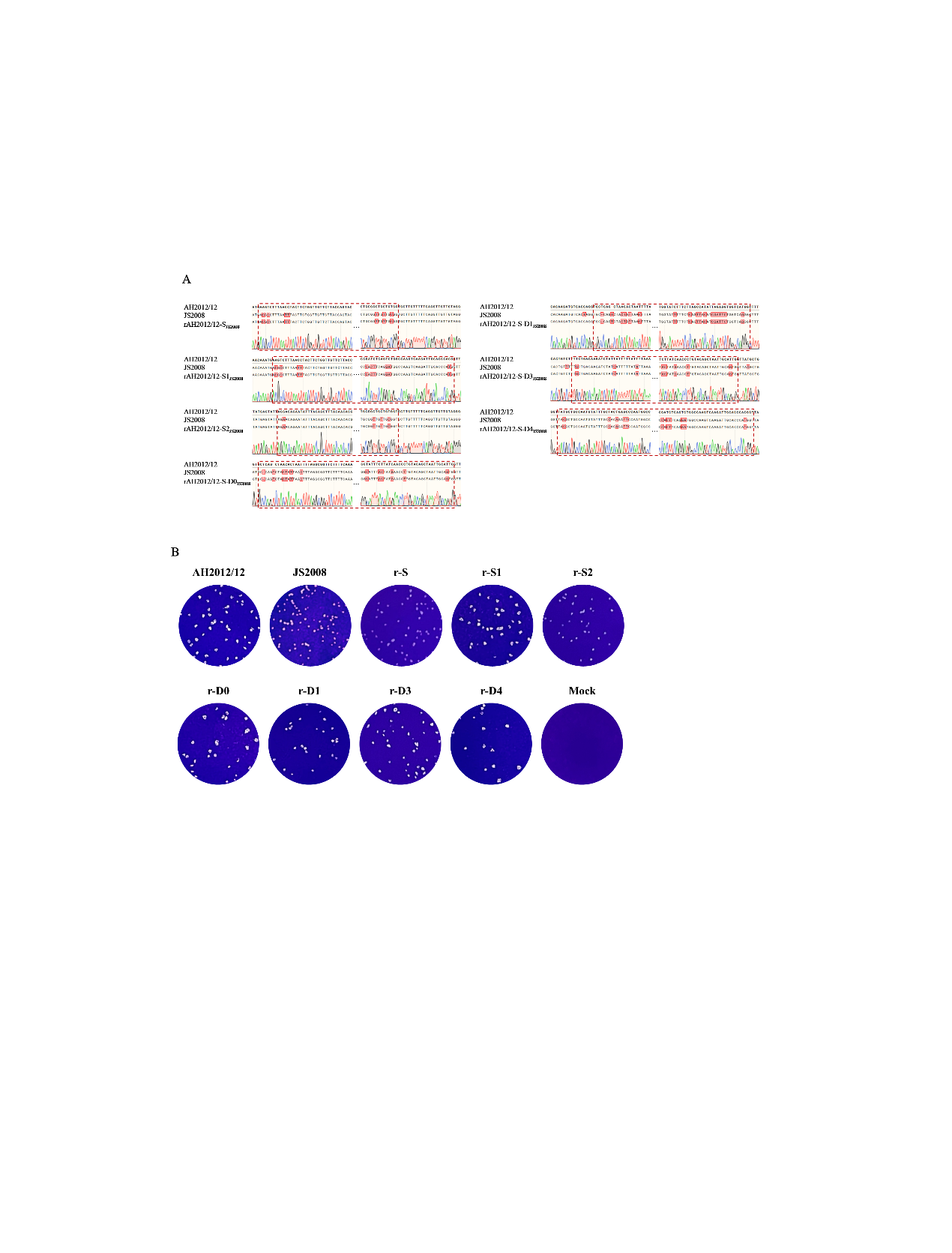


**Figure S1.** Construction of large-fragment substitution recombinant virus of the S gene. (A) Sequence identifications of eight recombinant plasmids with large-fragment substitutions in S gene segment. (B) Quantification of cell nuclei in syncytium formed by different recombinant viruses.


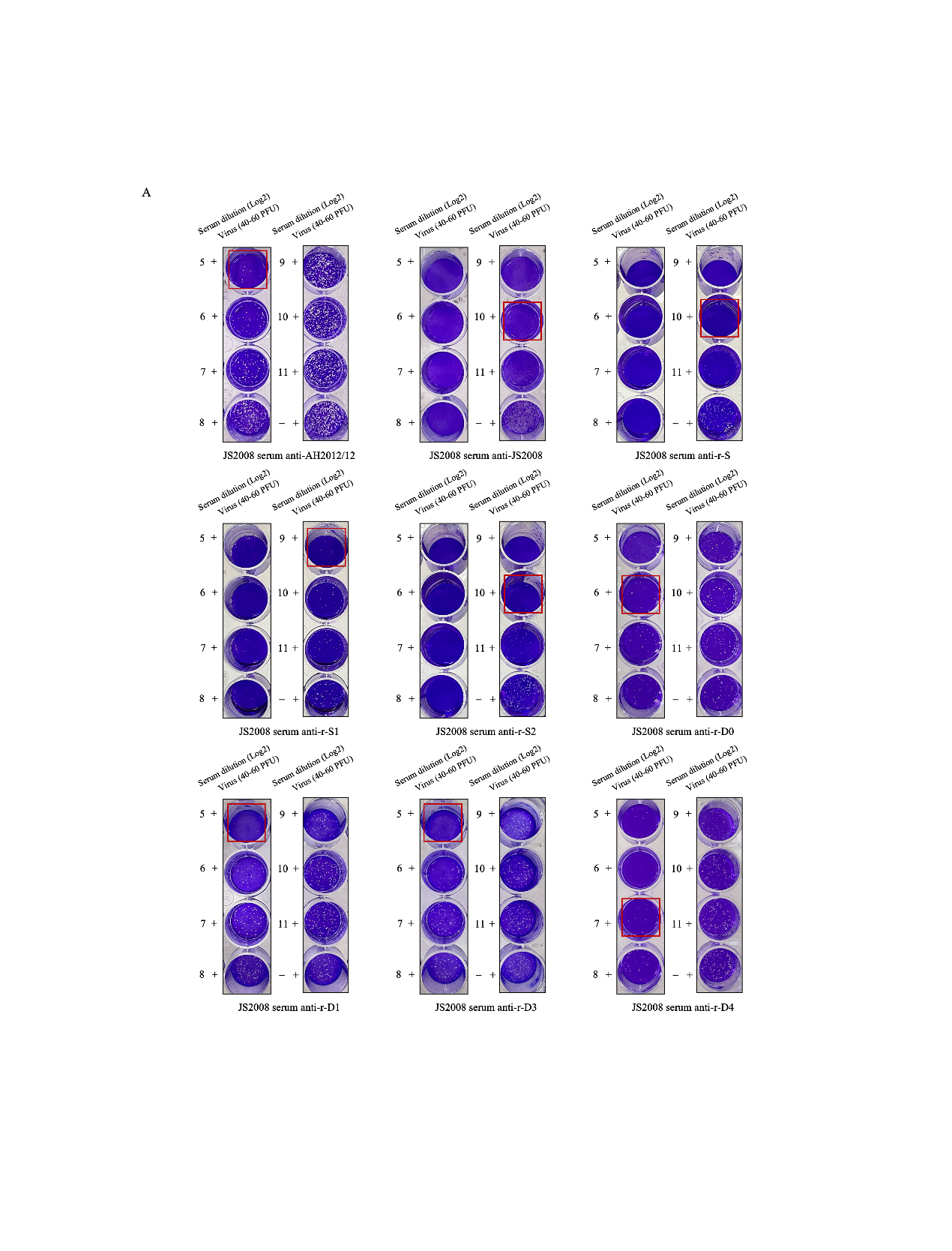


**Figure S2.** Plaque reduction neutralization test (PRNT) for large-fragment recombinant virus of the S gene. (A) PRNT of hyperimmune anti-JS2008 sera against each strain.


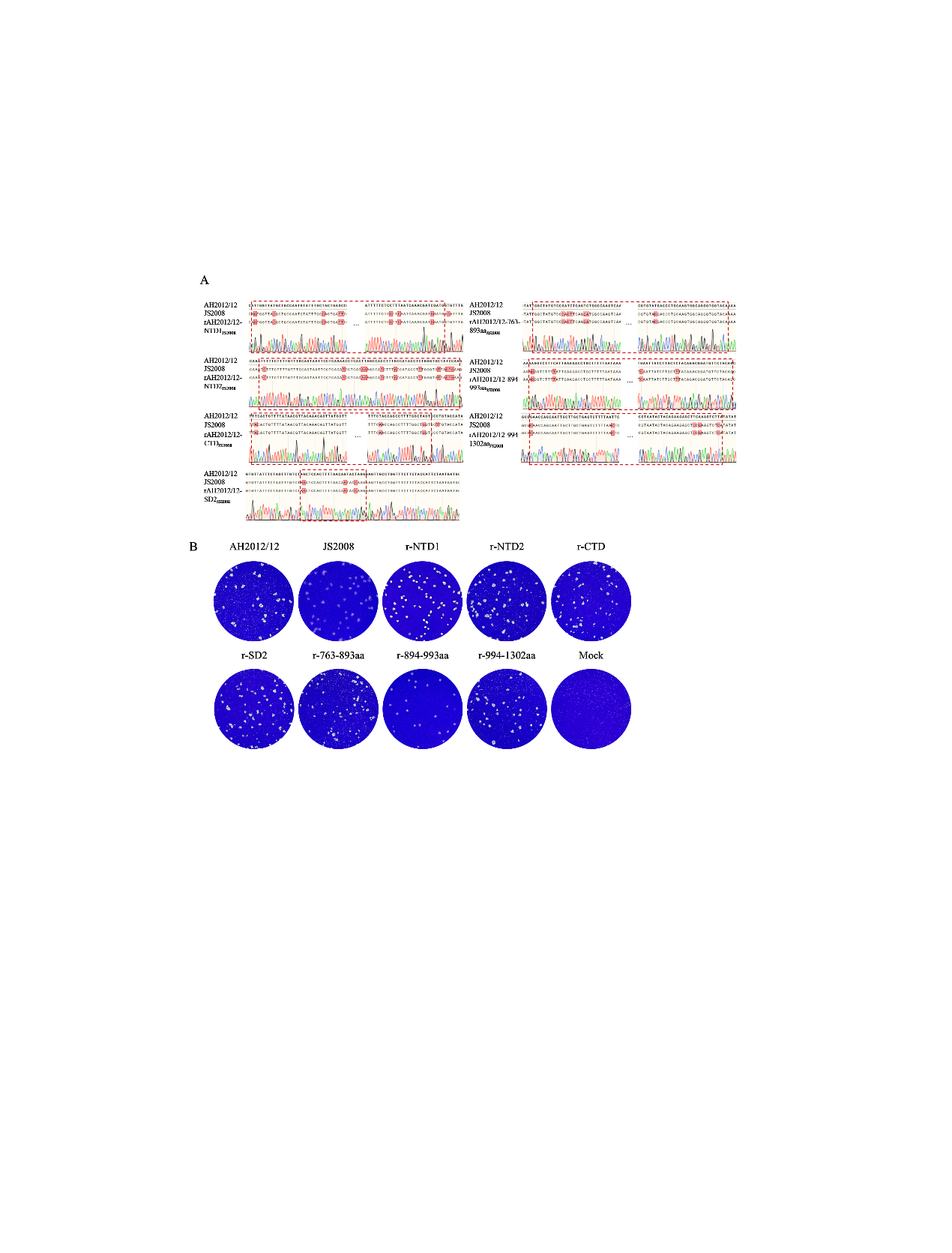


**Figure S3.** Construction of recombinant viruses with small-fragment substitution of the S gene. (A) DNA sequencing identification of eight recombinant plasmids with small-fragment substitutions in D4 and S2 gene segment. (B) Quantification of cell nuclei in syncytium formed by different recombinant viruses.

**
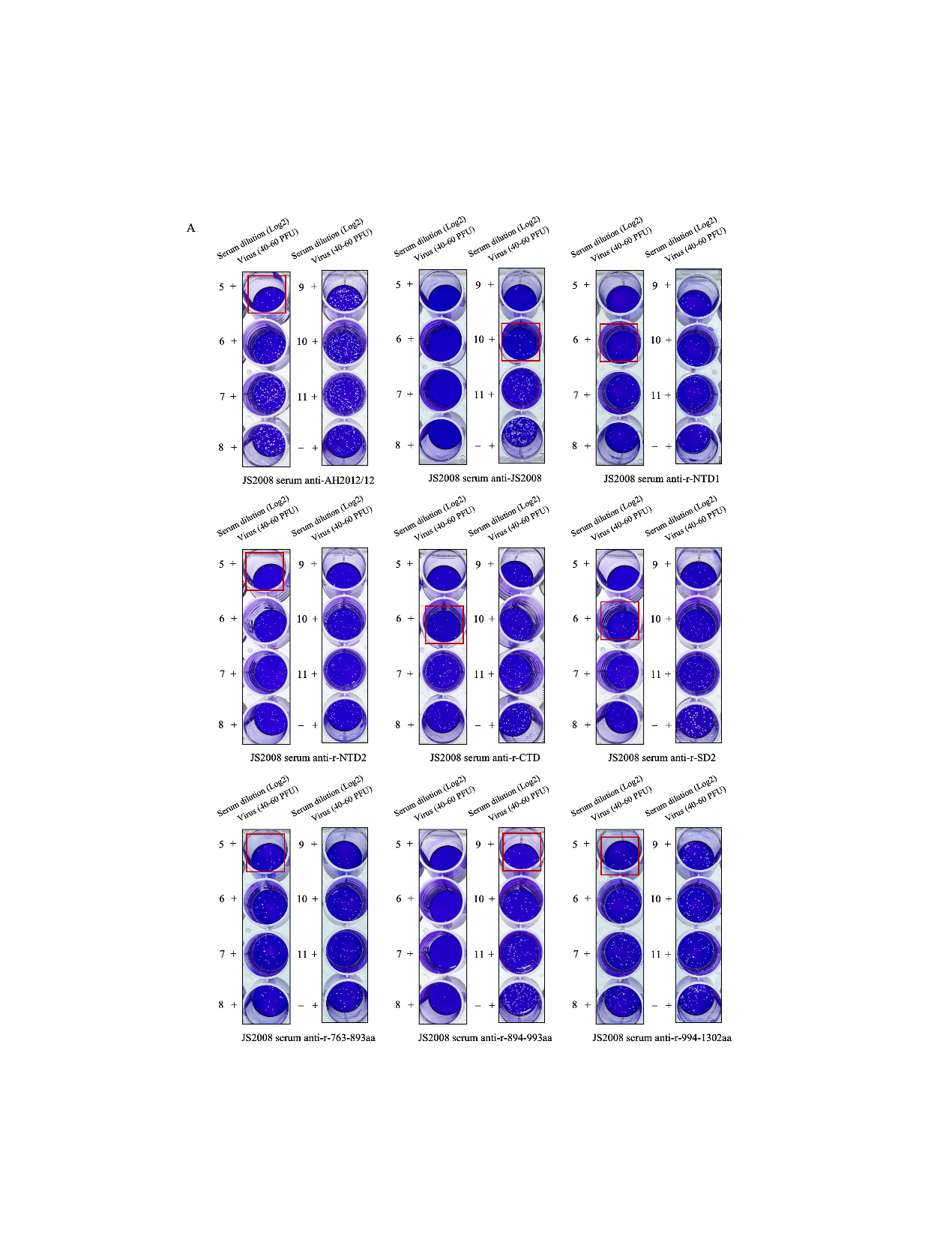
**

**Figure S4.** PRNT for small-fragment substitution recombinant virus of the S gene. (A) PRNT was performed to evaluate the neutralizing effects of rabbit-derived anti-JS2008 hyperimmune sera against each strain.

**
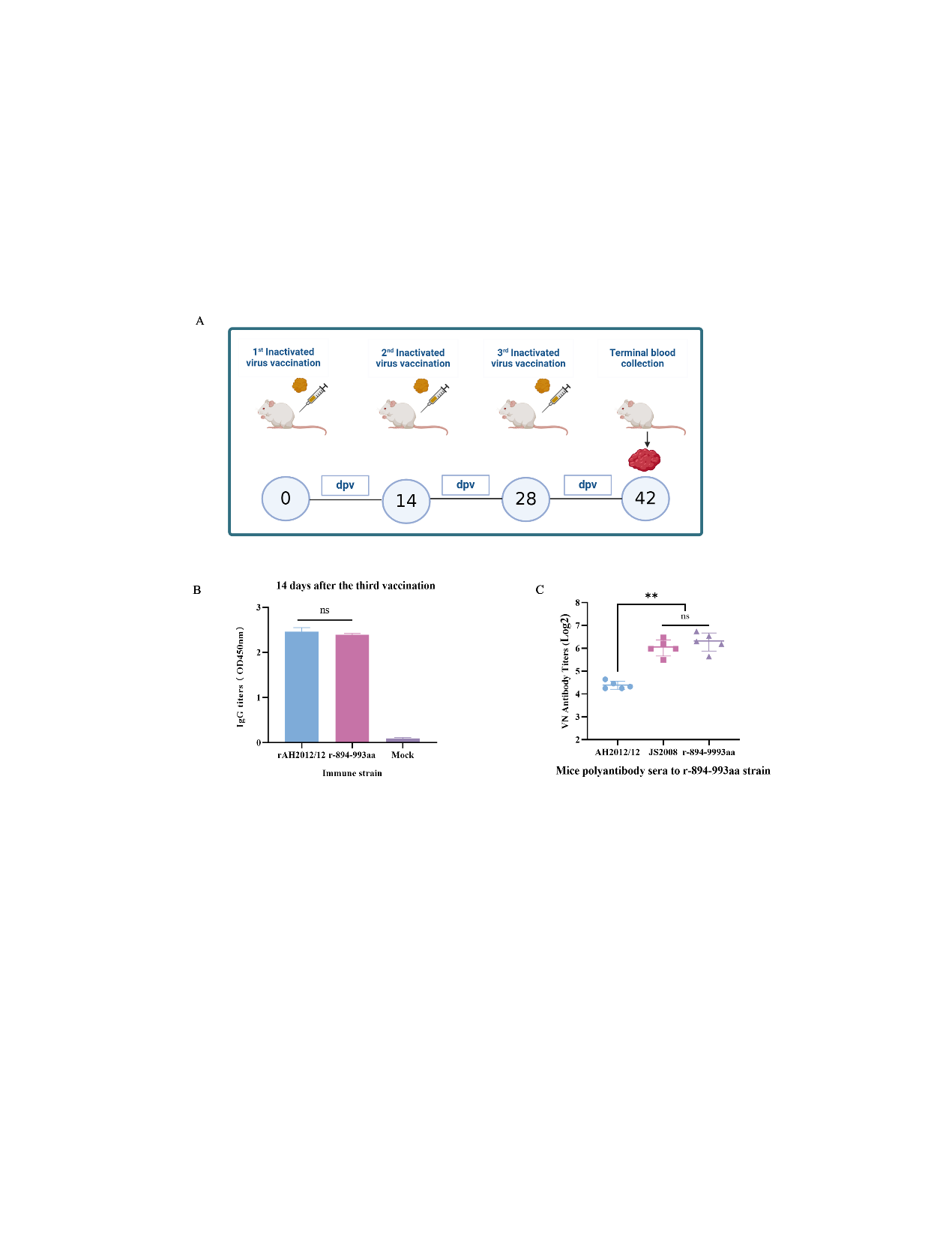
**

**Figure S5.** Immunogenicity and cross-neutralization activity assessment of the r-894-993aa recombinant virus. (A) Mice immunization procedures for rAH2012/12 and r-894-993aa. (B) Antibody titers in immune sera. Sera were collected 14 days after the third immunization from each group, and total IgG antibody titers against the PEDV S1 protein were measured. (C) Cross-neutralization results of mice-derived immune sera for r-894-993aa. Statistics: One-way ANOVA with multiple-comparison test (B and C). Statistical significance is indicated as follows: **p* < 0.05, ***p* < 0.01, ****p* < 0.001, *****p* < 0.0001.


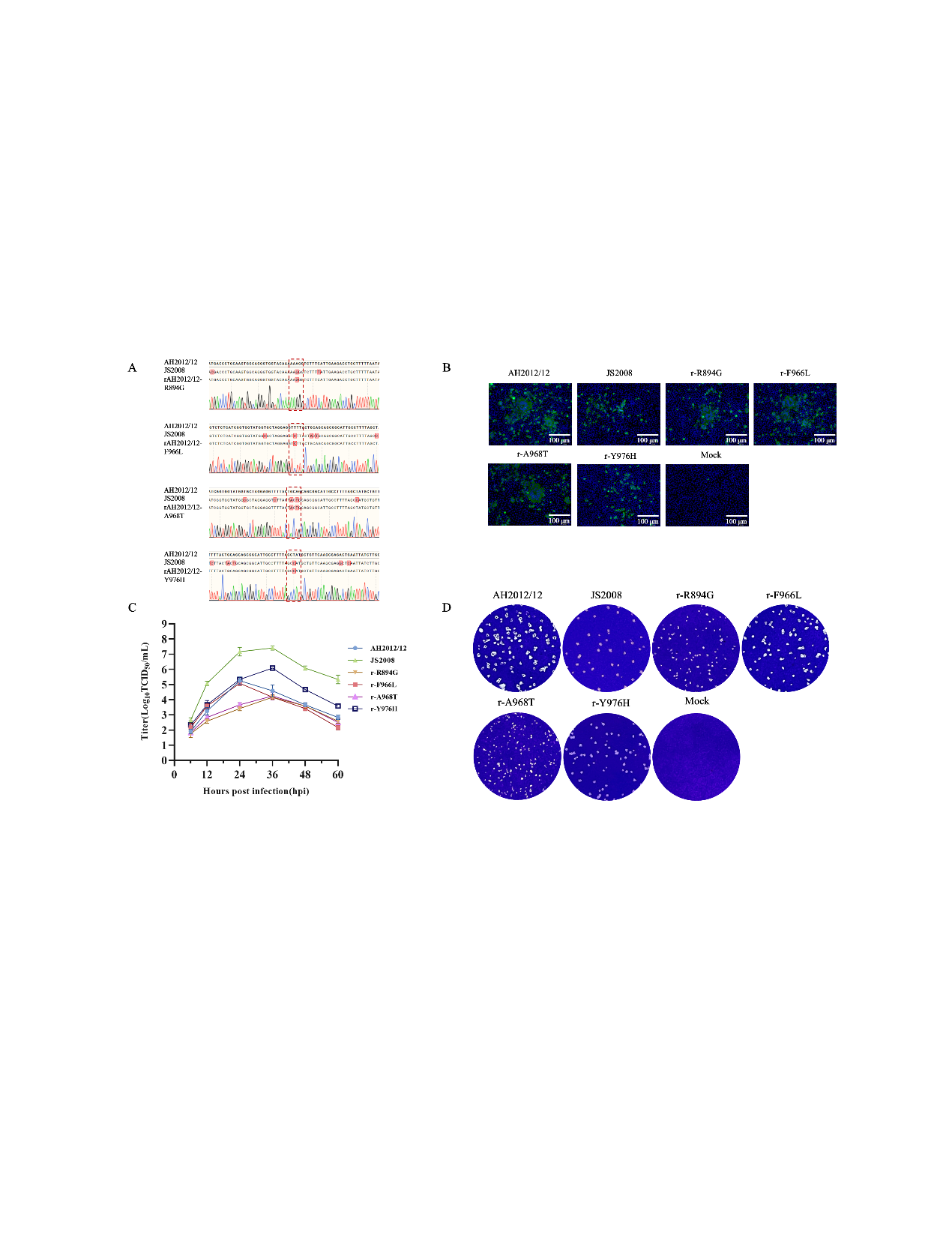


**Figure S6.** Construction of single-site mutant recombinant viruses of the S gene. (A) DNA sequencing identification of four recombinant plasmids with single-site mutation in 894-993 aa segment. (B) IFA, (C) growth curves and (D) plaque morphology of single-site mutant recombinant viruses.
